# Supplementary material for: Post-Cyclization Skeletal Rearrangements in Plant Triterpenoid Biosynthesis by a Pair of Branchpoint Isomerases
Source: J Am Chem Soc. 2023 Feb 23;145(9):5083–91. doi: 10.1021/jacs.2c10838 (PMC9999417; doi:10.1021/jacs.2c10838)
Supplement: Supplementary file 1 — ja2c10838_si_001.pdf [file ja2c10838_si_001.pdf]

Supporting Information for

Post-Cyclization Skeletal Rearrangements in Plant Triterpenoid Biosynthesis by a Pair of Branchpoint Isomerases

Ling Chuang[a]‡, Shenyu Liu[a]‡ and Jakob Franke[a][b]*

[a] Centre of Biomolecular Drug Research, Leibniz University Hannover, Schneiderberg 38, 30167 Hannover (Germany)

[b] Institute of Botany, Leibniz University Hannover, Herrenhäuser Str. 2, 30419 Hannover (Germany)

‡ These authors contributed equally.

* To whom correspondence should be addressed: jakob.franke@botanik.uni-hannover.de

Table of Contents

Table of Contents 2
Experimental Procedures 3
General chemical methods 3
General plant methods 3
Self-organizing map analysis and candidate selection 3
Construction of plasmids for transient expression in *Nicotiana benthamiana* 4
Transient expression of candidate genes in *Nicotiana benthamiana* 4
Metabolite extraction and LC-MS analysis 4
Synthesis of 7,8-epoxymelianol (9) 4
Purification of isomeliandiol (10) and protoglabretal (11) 5
Synthesis of isomeliandiol (10) 5
Isomeliandiol (10): 5
Yeast microsome preparation 5
In vitro enzyme assays 6
Phylogenetic analysis 6
References 44

Figures
Figure S1. Comparison of average expression patterns of the two clusters selected from SOM analysis to pathway gene expression patterns. 7
Figure S2. Candidate selection from self-organizing map (SOM) analysis of *Ailanthus altissima* transcriptome data. 7
Figure S3. Acid sensitivity of 7,8-epoxymelianol (9) to silica. 9
Figure S4. Time course showing degradation of 7,8-epoxymelianol (9) (peak at 6.6 min) in 50/50 MeCN/H_2O + 0.1% formic acid. Data shown are LCMS chromatograms (black: TIC; blue: EIC 511). 9
Figure S5. ^1H spectrum of 7,8-epoxymelianol (9) (C_6D_6, 298 K, 600 MHz). 15
Figure S6. ^{13}C spectrum of 7,8-epoxymelianol (9) (C_6D_6, 298 K, 151 MHz). 16
Figure S7. HSQC spectrum of 7,8-epoxymelianol (9) (C_6D_6, 298 K, 600 MHz). 17
Figure S8. HMBC spectrum of 7,8-epoxymelianol (9) (C_6D_6, 298 K, 600 MHz). 18
Figure S9. COSY spectrum of 7,8-epoxymelianol (9) (C_6D_6, 298 K, 600 MHz). 19
Figure S10. NOESY spectrum of 7,8-epoxymelianol (9) (C_6D_6, 298 K, 500 MHz). 20
Figure S11. ^1H NMR spectrum of isomeliandiol (10) ($CDCl_3$, 298 K, 500 MHz). 21
Figure S12. ^{13}C NMR spectrum of isomeliandiol (10) containing the C24/25 epoxide degradation products 12 and 13 ($CDCl_3$, 298 K, 126 MHz). The ^{13}C resonances of isomeliandiol (**10**) are shown in blue and key resonances of degradation products **12** and **13** are shown in grey. 22
Figure S13. HSQC spectrum of isomeliandiol (10) ($CDCl_3$, 298 K, 500 MHz). 23
Figure S14. HMBC spectrum of isomeliandiol (10) ($CDCl_3$, 298 K, 500 MHz). 24
Figure S15. COSY spectrum of isomeliandiol (10) ($CDCl_3$, 298 K, 500 MHz). 25
Figure S16. NOESY spectrum of isomeliandiol (10) ($CDCl_3$, 298 K, 500 MHz). 26
Figure S17. ^1H NMR spectrum of protoglabretal (11) ($CDCl_3$, 298 K, 500 MHz). 27
Figure S18. ^{13}C NMR spectrum of protoglabretal (11) ($CDCl_3$, 298 K, 126 MHz). 28
Figure S19. DEPT-135 spectrum of protoglabretal (11) ($CDCl_3$, 298 K, 100 MHz). 29
Figure S20. HSQC spectrum of protoglabretal (11) ($CDCl_3$, 298 K, 500 MHz). 30
Figure S21. HMBC spectrum of protoglabretal (11) ($CDCl_3$, 298 K, 500 MHz). 31
Figure S22. COSY spectrum of protoglabretal (11) ($CDCl_3$, 298 K, 500 MHz). 32
Figure S23. NOESY spectrum of protoglabretal (11) ($CDCl_3$, 298 K, 500 MHz). 33
Figure S24. Selected key HMBC and COSY correlations of 7,8-epoxymelianol (9). 34
Figure S25. Selected NOE correlations of 7,8-epoxymelianol (9). 34
Figure S26. Selected key HMBC and COSY correlations of isomeliandiol (10). 34
Figure S27. Selected NOE correlations of isomeliandiol (10). 35
Figure S28. Selected key HMBC and COSY correlations of protoglabretal (11). 35
Figure S29. Selected NOE correlations of protoglabretal (11). 35
Figure S30. *In vitro* activity of AaCYP88A154, AaISM1 and AaISM2 produced in *Saccharomyces cerevisiae* microsomes is consistent with activity in *Nicotiana benthamiana*. 41
Figure S31. Overview over common C-8,7 sterol isomerase (8,7SI) reactions in primary metabolism. 42
Figure S32. Overview over other isomerase-catalyzed reactions in plant specialized metabolism.[61–65] 43

Tables
Table S1. Sequences of the primers used in this study. The start and stop codons are marked in red. For In-Fusion cloning, the overlapping sequences to the vector are marked in blue. For Golden gate cloning, BsaI restriction sites are marked in green, and the cutting site is indicated with a slash 8
Table S2. Conditions for extraction and flash chromatography purification of isomeliandiol (10) and protoglabretal (11). 10
Table S3. ^1H and ^{13}C NMR data of 7,8-epoxymelianol (9) (C_6D_6, 298 K, 600 MHz). 11
Table S4. ^1H and ^{13}C NMR data of isomeliandiol (10) obtained as a mixture of epimers ($CDCl_3$, 298 K, 500 MHz). 12
Table S5. Partial ^1H and ^{13}C NMR data of degradation products 12 and 13 present in NMR samples of isomeliandiol (10) ($CDCl_3$, 298 K, 500 MHz). 13
Table S6. ^1H and ^{13}C NMR data of protoglabretal (11) ($CDCl_3$, 298 K, 500 MHz). 14
Table S7. Representative examples of apoprotolimonoids previously isolated from Meliaceae, Rutaceae, and Simaroubaceae plants out of ca. 120 structurally related natural products listed in Reaxys. Stereochemistry is shown as reported in the literature. 36
Table S8. Representative examples of glabretanes previously isolated from Meliaceae, Rutaceae, and Simaroubaceae plants out of ca. 110 structurally related natural products listed in Reaxys. Stereochemistry is shown as reported in the literature. 38

Experimental Procedures

General chemical methods

NMR spectra were recorded using Bruker Ultrashield 400, Ultrashield 500 or Ascend 600 MHz spectrometers operating at 400, 500 and 600 MHz for ^1H NMR and at 100, 126 and 151 MHz for ^{13}C NMR. $CDCl_3$ and C_6D_6 were used as solvents. Chemical shifts were referenced relative to the residual solvent signals ($CDCl_3$: δ_H = 7.26 ppm, δ_C = 77.16 ppm; C_6D_6: 7.16 ppm, δ_C = 128.06 ppm) and expressed in δ values (ppm), with coupling constants reported in Hz. Analysis was conducted with TopSpin (Version 4.0.6) or MestReNova (Version 14.2).
HRMS measurements were carried out on a Waters Alliance 2695 HPLC coupled to a Micromass LCT Premier mass spectrometer.
Analytical and semipreparative LCMS analyses were performed on an Agilent Infinity II 1260 system consisting of a G7167A autosampler, G7116A column thermostat, G7111B quaternary pump, G7110B make-up pump, G7115A diode array detector, G1364F fraction collector, and G6125B single quadrupole mass spectrometer equipped with an ESI source (positive mode, 4000 V, 12 L/min drying gas, 350 °C gas temperature). The columns and gradients used are described below.
Automated flash chromatography was performed on a Biotage Isolera One with the stationary phases, solvents and gradients described below.
Melianol as a reference compound and substrate was obtained by transient expression in *N. benthamiana* followed by purification as described previously.[1] All other reagents were purchased from Sigma-Aldrich, Fisher Scientific and Carl Roth. All reagents were directly used without further purification unless mentioned otherwise. Dry solvents were obtained from Acros Organics and stored under N_2 with molecule sieve (3 Å).

General plant methods

Ailanthus altissima plants were grown from seedlings as described previously.[1] Work with *Ailanthus altissima* in our research group is granted by permit DE-NI-2019-001 (NLWKN, Niedersächsischer Landesbetrieb für Wasserwirtschaft, Küsten- und Naturschutz) in addition to regulation (EU) No. 1143/2014. *Nicotiana benthamiana* LAB strain[2] was grown from seeds in a greenhouse with 11 to 16 hours illumination per day and at a temperature between 21 °C to 23 °C as described previously.[3]

Self-organizing map analysis and candidate selection

Self-organizing map (SOM) analysis was based on expression data from our previously published de novo transcriptome data, covering 14 different tissue samples from *Ailanthus altissima* seedlings or 3-year-old trees.[1] Shortly, raw reads from RNA-Seq were assembled *de novo* with Trinity 2.11.0,[4] and contig expression was determined by Salmon 1.3.0[5] and normalized using the TMM method.[6] The SOM analysis was conducted in R version 4.1.2 using the kohonen package,[7] using a slightly adjusted version of the script reported by Payne *et al*.[8] Cluster quality was calculated based on the within-node distance and inter-nodal distance as described in Payne *et al.*;[8] high cluster quality corresponds to low within-node and low inter-nodal distance. Broad grey lines additionally indicate neighboring clusters with low inter-nodal distance (25% quantile of all).
The node containing transcripts for the previously reported quassinoid pathway genes *AaCYP71CD4* and *AaCYP71BQ17* was manually screened to select gene candidates. For the 695 contigs in the node, protein sequences were deduced using TransDecoder 5.3.0[9] and protein domains annotated using a search with hmmscan 3.2[10] against the Pfam-A database.[11] Candidates were then extracted by filtering the 695 co-expressed contigs for Pfam, blastx and blastn hits containing the keywords p450, oxidoreductase, oxidase, oxygenase, reductase, dehydrogenase (138 hits). The further filtering steps are shown in Figure S2. An analogous strategy was used for isomerase selection (keywords: isomerase, epimerase, mutase) as shown in Figure S2.

Construction of plasmids for transient expression in *Nicotiana benthamiana*

Primers for the amplification of full coding sequences were designed based on transcript sequences of candidate genes. The primer sequences are listed in Table S1. The insert sequences were amplified with SuperFi II polymerase (Thermo Fisher Scientific) from cDNA of seedling root. Amplicons were cloned either into the vector pEAQ-HT[12] by In-Fusion HD cloning (Takara Bio), or into the vector pHREAC[13] as described previously by Golden Gate cloning.[3] Cloned sequences were confirmed by Sanger sequencing. Coding sequences of genes *AaCYP88A154*, *AaISM1*, and *AaISM2* identified in this study were deposited in GenBank under the accession numbers ON942227-ON942229.

Transient expression of candidate genes in *Nicotiana benthamiana*

For agroinfiltration into *N. benthamiana*, a procedure described earlier by us was used.[3] In short, the plasmids containing gene candidates for transient expression were transformed into *Agrobacterium tumefaciens* GV3101 by electroporation. *A. tumefaciens* strains were precultured for 2 days at 28 °C in LB + 25 µg/mL gentamicin + 50 µg/mL rifampicin + 50 µg/mL kanamycin. After the preculture, *A. tumefaciens* cells were harvested and resuspended in MMA infiltration buffer (10 mM $MgCl_2$, 10 mM 2-(*N*-morpholino)ethanesulfonic acid (MES), 100 µM acetosyringone). Strains carrying candidate genes were mixed with *A. tumefaciens* strains carrying *AstHMGR* (KY284573),[14] *AaOSC2* (ON595696), *AaCYP71CD4* (ON595698), and *AaCYP71BQ17* (ON595699) (all strains at OD_{600} 0.1) prior to syringe infiltration into the abaxial side of *Nicotiana benthamiana* leaves. After infiltration, plants were maintained in a greenhouse until further analysis. For screening, at least three biological replicates were used for each combination.

Metabolite extraction and LC-MS analysis

Around 10 mg dry weight of infiltrated leaves were used for metabolite extraction. Infiltrated leaves were harvested 7 days after infiltration. Five leaf disks were harvested using cork-borer no. 5 (10mm) and lyophilized before extraction with 800 µL 90% methanol. After removal of solid debris by centrifugation, the crude extract was directly used for LC-MS analysis. Samples were separated on a C18 column (Poroshell 120 EC-C18, dimensions: 100 × 4.6 mm, particle size: 2.7 µm) using an LCMS system from Agilent (Agilent 1260 II Infinity, Santa Clara, CA, USA). The column temperature was set at 50 °C. As mobile phase, solvent A (water with 0.1%(v/v) formic acid) and solvent B (acetonitrile with 0.1%(v/v) formic acid) were used. Separation was achieved using the following gradient at a flow rate of 1 mL/min: 0-1 min, 10-40% B; 1-11 min, 40-90% B; 11-13 min, 90% B; 13-13.1 min, 90-10% B; 13.1-15 min, 10% B.

Synthesis of 7,8-epoxymelianol (9)

m-CPBA was purified before use following Kazmaier's report.[15] The exact concentration was determined by NMR. Melianol (11 mg, 23 µmol, 1 eq.) was charged in a flame-dried Schlenk round bottom flask under N_2 atmosphere and dissolved in dry DCM (6 mL). The reaction mixture was cooled to 0 °C with an ice-water bath, and *m*-CPBA (14 mg, 85% purity, 70 µmol, 3 eq.) in dry DCM (4 mL) was added dropwise over 10 mins at 0 °C. When the addition of *m*-CPBA was complete, the reaction mixture was allowed to warm to room temperature and stirred for additional 3 hrs. After that, 5 mL Na_2SO_3 solution (5% w/v) was added into the reaction mixture. The biphasic mixture was stirred vigorously for 5 mins. The organic layer was then collected, washed with sat. $NaHCO_3$ (5 mL), water (5 mL) and brine (5 mL) sequentially and dried over Na_2SO_4. The solvent was removed with a gentle stream of of N_2 to give 7,8-epoxymelianol (**9**) (10 mg, 90%) as white powder. No further purification was performed. For the success of this reaction, a low concentration of substrate proved critical to minimize overoxidation and rearrangements.

7,8-Epoxymelianol (9):
HR-ESI-MS: $[M+Na]^+$ = 511.3409 (calcd. For $C_{30}H_{48}O_4Na^+$ 511.3399). ^1H and ^{13}C NMR data see Table S3.

Purification of isomeliandiol (10) and protoglabretal (11)

For compound isolation, *N. benthamiana* plants were vacuum infiltrated in a 9.2 L ROTILABO desiccator (Carl Roth, Karlsruhe, Germany) connected to a MZ 2 NT membrane pump (Vacuubrand, Wertheim, Germany) at 30 mbar for 1.5 min. 60 plants were each used for purification of the AaCYP88A154 and AaISM2 products.
Leaves were harvested 7 days post infiltration and lyophilized for 2-3 days until the dry weight remained constant. The crude plant material was ground at room temperature in a blender to powder form and extracted with ethyl acetate (AaCYP88A154 product) or 90/10 MeOH/H_2O (AaISM2 product), filtered and concentrated *in vacuo*. The crude extracts were purified by successive rounds of flash chromatography (Biotage Isolera One) as described in Table S2. This process yielded 2 mg isomeliandiol (**10**) (0.12 mg / g dry weight) as a white powder, and 22 mg protoglabretal (**11**) (1.3 mg / g dry weight) as a yellow solid.

Synthesis of isomeliandiol (10)

In a glass vial, 7,8-epoxymelianol (**9**) (2.0 mg, 4.1 µmol) was dissolved in 50% acetonitrile-water solution (2 ml) and formic acid (2 µl) was added. The solution was allowed to stir at room temperature. The rearrangement was monitored by LC-MS and completed in 4 hours. The solution was then concentrated *in vacuo* and purified by semipreparative HPLC as described below; fractions were collected by time-based mode. After lyophilization to remove the solvent, isomeliandiol (0.8 mg, 1.6 µmol, 40%) was obtained as colorless solid.
Semipreparative HPLC for separation of the crude reaction solution was performed on a C18 column (Phenomenex Kinetex, 5 µm, 100 Å, 250 × 10 mm). The column temperature was set to 40 °C. Separation was achieved with the following gradient using a combination of solvent A (water with 0.1%(v/v) formic acid) and solvent B (acetonitrile with 0.1%(v/v) formic acid) at 5 mL/min: 0-10 min, 40%-100% B; 10-12 min, 100% B; 12-12.5 min, 100-40% B; 12.5-15 min, 40% B. The fraction between 6.63 and 6.89 min contained isomeliandiol (**10**) and was collected.

Isomeliandiol (10):

HR-ESI-MS: $[M+Na]^+$ = 511.3389 (calcd. For $C_{30}H_{48}O_5Na^+$ 511.3399). ^1H and ^{13}C NMR data see Table S4.

Protoglabretal (11):

HR-ESI-MS: $[M+Na]^+$ = 511.3380 (calcd. For $C_{30}H_{48}O_5Na^+$ 511.3399). ^1H and ^{13}C NMR data see Table S6.

Yeast microsome preparation

AaCYP88A154, *AaISM1*, and *AaISM2* were cloned into the yeast expression vector pYES2 (Thermo Fisher Scientific) using the In-Fusion kit (Takara Bio). Insert sequences were confirmed by Sanger sequencing. pYES2-AaISM1, pYES2-AaISM2 and pYES2 (EV) were transformed to yeast strain Y10000 (BY4742, S288C isogenic yeast strain: MATα; his3Δ1; leu2Δ0; lys2Δ0; ura3Δ0) using the LiAc/SS carrier DNA/PEG method.[16] pYES2-AaCYP88A154 and pYES2 (EV) were transformed to yeast strain WAT11U (MATα (leu2-3,112; trp1-1; can1-100; ura3-1; ade2-1; his3-11,15)[17] using the same method. Transformed colonies were checked by colony PCR. The transformed yeast strains were grown in synthetic medium without uracil (0.17% (w/v) yeast nitrogen base without amino acids (Sigma-Aldrich), 0.077% (w/v) yeast synthetic drop-out medium supplements without uracil (Sigma-Aldrich) and additional 0.5% (w/v) ammonium sulfate) with 2% glucose at 30 °C at 180 rpm for two days and then transferred to the same synthetic medium without uracil with 2% galactose to induce gene expression and grown at 30 °C at 180 rpm for another two days. Yeast cells were harvested by centrifugation at 10,000 × *g* at 4 °C for 10 minutes, washed with TEK buffer (50 mM Tris, pH 7.4, 1 mM EDTA, 0.1 M KCl) and then lysed with a French press in TEB buffer (50 mM Tris, pH 7.4, 1 mM EDTA, 0.6 M sorbitol). Microsomes were then isolated by ultracentrifugation at 100,000 × *g* at 4 °C for 1 hour, resuspended in TEG buffer (50 mM Tris, pH 7.4, 1 mM EDTA, 20% glycerol), and stored at −80 °C prior to enzyme assays.

In vitro enzyme assays

Yeast microsome assays were performed in 200 µL total volume containing reaction buffer (50 mM Tris-HCl, pH 7.5, 2 mM $MgCl_2$, 1 mM EDTA, 2 mM 2-mercaptoethanol, 5% glycerol and 0.1% Tween 80), 100 µM of the substrate, 500 µM of NADPH for reactions involving AaCYP88A154, and 50 µL of yeast microsomes (25 µL each if mixing microsomes from two strains). The substrates melianol (**8**), 7,8-epoxymelianol (**9**), and isomeliandiol (**10**) were dissolved in ethanol at 200 mM stock concentration. The reactions including AaCYP88A154 were conducted at 30 ˚C, 200 rpm for 16 hours. The reactions of AaISM2 alone were conducted at 30 ˚C, 200 rpm for 90 minutes. The reactions were quenched by adding 800 µL of methanol followed by centrifugation at 14,000 × *g* at room temperature for 5 minutes to remove the precipitated proteins. After concentrating *in vacuo* at 40 mbar for 1.5 hours in a speed vac, the remainder was dissolved in 90% methanol for LC-MS injection. The same LC-MS method as described above for *Nicotiana benthamiana* experiments was used.

Phylogenetic analysis

To identify homologues of C-8,7 sterol isomerase (8,7SI), AaISM1 and AaISM2 in Sapindales plants, the peptide sequences of characterized *Arabidopsis thaliana* 8,7SI (AF030357.1)[18], AaISM1 and AaISM2 were used as reference for blastp searches against publicly available databases. For orange (*Citrus sinensis*) and pomelo (*Citrus grandis*) from Rutaceae family, the peptide sequences were downloaded from Citrus Pan-genome to Breeding Database (CPBD) v2.0 and v1.0 respectively[19,20]. The sequences of heiyouchun (*Toona sinensis*) from Meliaceae were downloaded from China National GeneBank DataBase (CNGBdb) under the project ID CNP0000958[21]. Sequences of mango (*Mangifera indica*) from Anacardiaceae family were kindly provided by Wang *et al.*,[22] and yangbi mapel (*Acer yangbiense*) data in Sapindaceae family were downloaded from GigaDB [23]. For plants outside of Sapindales, the model plants *Arabidopsis thaliana* and *Nicotiana benthamiana* were chosen and their sequences were downloaded from TAIR (Araport 11) and Solgenomics (genome sequence v1.0.1) respectively. Unique full length peptide sequences with the best E-values and coverage resulting from the blastp search were chosen for alignment and tree construction.

In addition to At8,7SI, characterized 8,7SI from other plants and non-plants were included as reference. For plants, Zm8,7SI (AY533175.1) from corn (*Zea mays*) [24] and a putative 8,7SI (AK059848.1) from *Oryza sativa* validated at transcript level were included[24,25]. For organisms from other kingdoms, Rn8,7SI(AF071501.1) from rat (*Rattus norvegicus*) [26], Mm8,7SI (X97755.1) from house mouse (*Mus musculus*) [27], Cp8,7SI (Q60490) from Guinea pig (*Cavia porcellus*), Hs8,7SI (NP_006570) from human (*Homo sapiens*) [28,29], and a fungal 8,7SI from *Thermothelomyces thermophilus* (XP_003665660)[30] were included.

A multiple sequence alignment of all isomerases was generated using Clustal Omega 1.2.2[31–33]. Then, a phylogenetic tree was constructed using the maximum likelihood method with PhyML 3.3.20180621[34] in Geneious 2021.1.1.

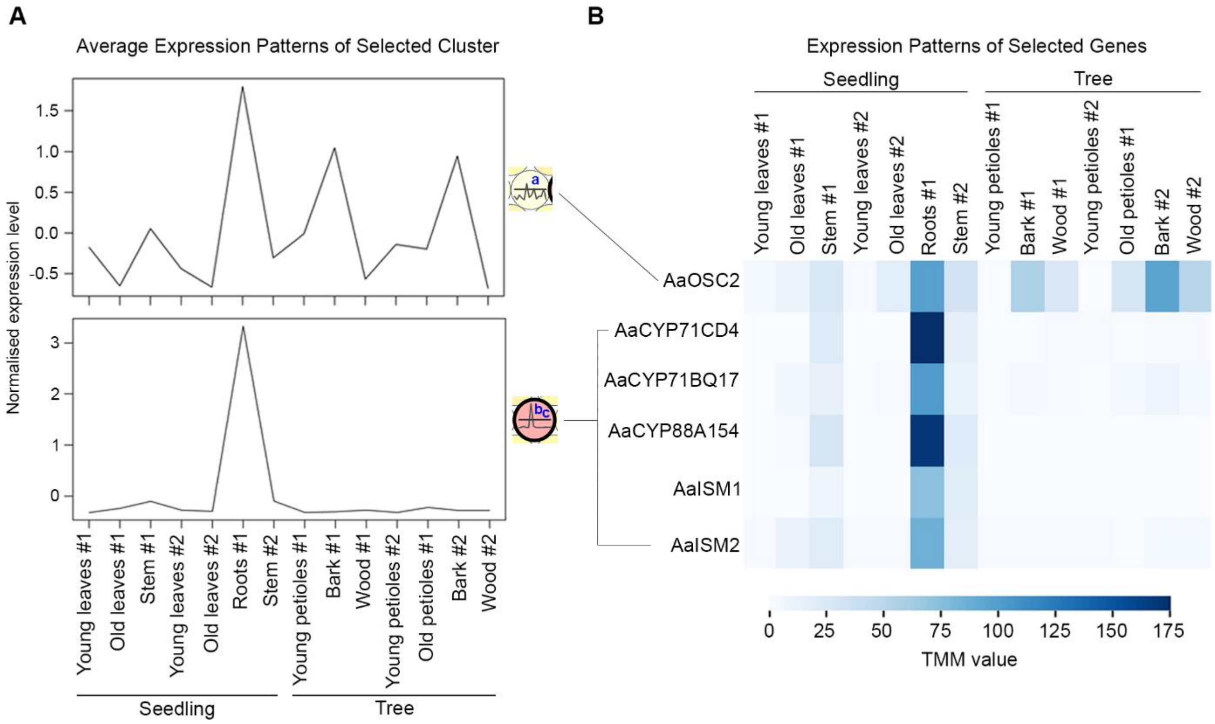

Figure S1. Comparison of average expression patterns of the two clusters selected from SOM analysis to pathway gene expression patterns. (A) Average expression pattern of the cluster containing *AaOSC2* (199 contigs in total) (top), and of the cluster containing *AaCYP71CD4* and *AaCYP71BQ17* (695 contigs in total). (B) Expression patterns of verified quassinoid pathway genes as a heat map. The expression level is represented by trimmed mean of M-values (TMM) values in the different tissues of *Ailanthus altissima.* Lines between (A) and (B) connect the genes with the corresponding clusters from SOM analysis.

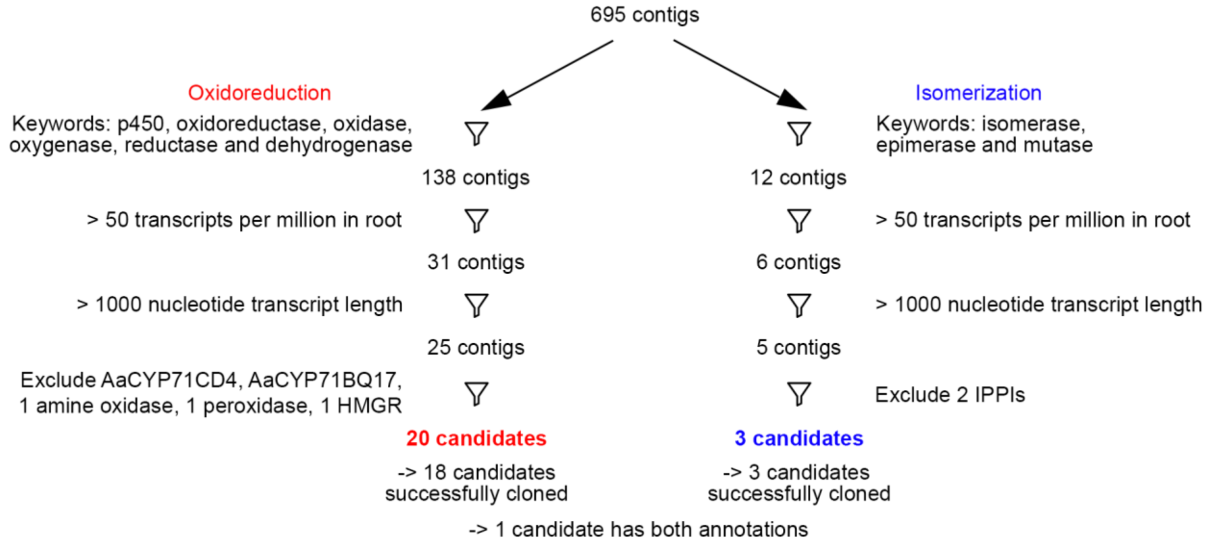

Figure S2. Candidate selection from self-organizing map (SOM) analysis of *Ailanthus altissima* transcriptome data. The 695 contigs found in the cluster with *AaCYP71CD4* and *AaCYP71BQ17* were first filtered by keywords in their blastn, blastx and pfam annotations. This results in 138 contigs for contigs with oxidoreduction annotations and 12 contigs with isomerization annotations. Among them, 5 contigs were found with both annotations. Contigs with low expression level in root and insufficient nucleotide length were then excluded. Also, candidates with unrelated functions based on their full annotations as well as the two reference CYP450s were manually removed. The resulting contigs were selected as candidates. Abbreviations: isopentenyl-diphosphate delta isomerase (IPPI) and 3-hydroxy-3-methylglutaryl-CoA reductase (HMGR).

Table S1. Sequences of the primers used in this study. The start and stop codons are marked in red. For In-Fusion cloning, the overlapping sequences to the vector are marked in blue. For Golden gate cloning, BsaI restriction sites are marked in green, and the cutting site is indicated with a slash

Primer name	Primer sequence	Vector
AaOSC2_Fw	CACCACAGGTCTCG/AAAAATGTGGAGGCTTAAGATTGCAGA	pHREAC
AaOSC2_Rv	CACCACAGGTCTCG/AGCGTCAATTAGGCAATGGAACTTTCCT	pHREAC
AaCYP71CD4_Fw	GCCCAAATTCGCGACCGGATGATGGAGCTACAGCTTGA	pEAQ-HT
AaCYP71CD4_Rv	CAGAGTTAAAGGCCTCGATCACGGATCGTAAGGAGTGG	pEAQ-HT
AaCYP71BQ17_Fw	GCCCAAATTCGCGACCGGTTGAGAACAAAATTGCCAATGGA	pEAQ-HT
AaCYP71BQ17_Rv	CAGAGTTAAAGGCCTCGATCACTTCTGGAAAGGAATATGAGTG	pEAQ-HT
AaCYP88A154_Fw	GCCCAAATTCGCGACCGGATGCTAAGAAACTCAGACATCA	pEAQ-HT
AaCYP88A154_Rv	CAGAGTTAAAGGCCTCGATCATTTGAGCTTAACGACTTTTGC	pEAQ-HT
AaISM1_Fw	CACCACAGGTCTCG/AAAAATGAGCAATTCGTATATGCCCA	pHREAC
AaISM1_Rv	CACCACAGGTCTCG/AGCGTCAGCAAACTTTGGCCTTCT	pHREAC
AaISM2_Fw	CACCACAGGTCTCG/AAAAATGAGCAACCATCCCTATTCTCC	pHREAC
AaISM2_Rv	CACCACAGGTCTCG/AGCGTCAGTAGAATTTGGCTTTCTTCTGA	pHREAC
AaCYP88A154_Y_Fw	CACTATAGGGAATATTAAGCTATGCTAAGAAACTCAGACATCA	pYES2
AaCYP88A154_Y_Rv	CATGATGCGGCCCTCTAGTCATTTGAGCTTAACGACTTTTGC	pYES2
AaISM1_Y_Fw	CACTATAGGGAATATTAAGCTATGAGCAATTCGTATATGCCCA	pYES2
AaISM1_Y_Rv	CATGATGCGGCCCTCTAGTCAGCAAACTTTGGCCTTCT	pYES2
AaISM2_Y_Fw	CACTATAGGGAATATTAAGCTATGAGCAACCATCCCTATTCTCC	pYES2
AaISM2_Y_Rv	CATGATGCGGCCCTCTAGTCAGTAGAATTTGGCTTTCTTCTGA	pYES2

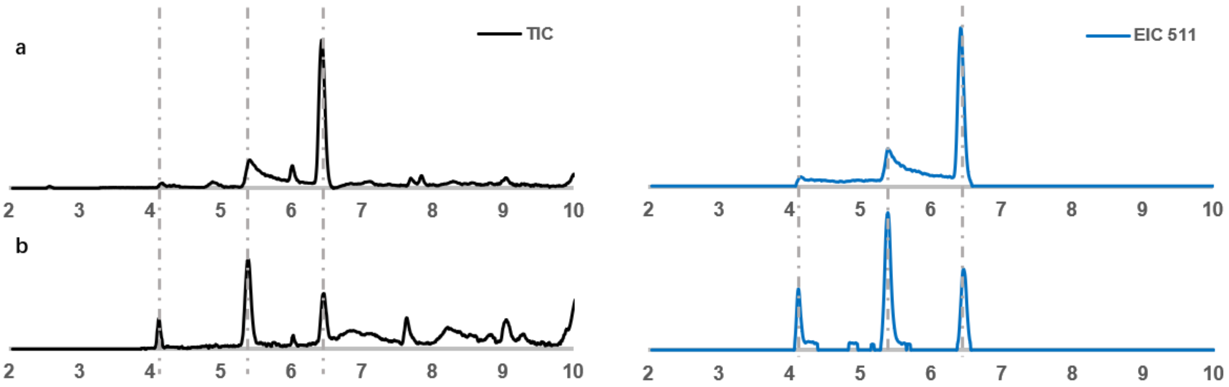

Figure S3. Acid sensitivity of 7,8-epoxymelianol (9) to silica. Sample composition of the crude reaction product from melianol epoxidation with *m*-CPBA before (a) and after (b) attempts to purify the 6.6 min peak corresponding to 7,8-epoxymelianol (**9**) by normal phase column chromatography on silica. Data shown are LCMS chromatograms (black: TIC; blue: EIC 511).

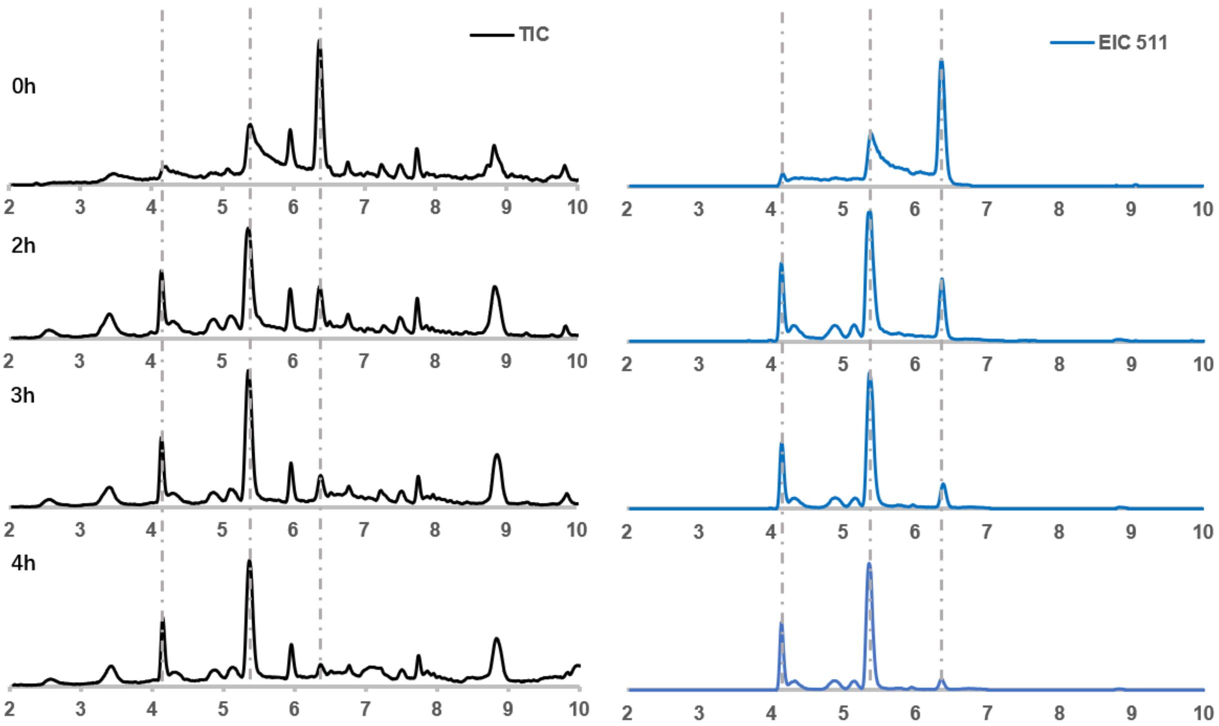

Figure S4. Time course showing degradation of 7,8-epoxymelianol (9) (peak at 6.6 min) in 50/50 MeCN/H_2O + 0.1% formic acid. Data shown are LCMS chromatograms (black: TIC; blue: EIC 511).

Table S2. Conditions for extraction and flash chromatography purification of isomeliandiol (10) and protoglabretal (11).

**Isomeliandiol (10)**	**Fresh weight:**	82.27 g	**Dry weight:**	16.06 g
	**Extraction solvent:**	Ethyl acetate	**Crude extract:**	1.18 g
	**Column**	**Solvents**	**Gradient**	**Yield**
	SNAP KP-Sil 50 g	A: Petroleum ether<br>B: Ethyl acetate	10-100% (10 CV)<br>100% (3 CV)	164 mg
	Sfär C18 D 12 g	A: Water<br>B: Acetonitrile	40-100% (11 CV)	27 mg
	SNAP Ultra 10 g	A: Petroleum ether<br>B: Ethyl acetate	30% (2 CV)<br>30-60% (3 CV)<br>60% (7 CV)<br>60-80% (3 CV)<br>80% (3 CV)<br>80%-100 (3 CV)	9 mg
	Sfär C18 D 12 g	A: Water<br>B: Acetonitrile	60-100% (11 CV)	2 mg
**Protoglabretal (11)**	**Fresh weight:**	114.95 g	**Dry weight:**	17.59 g
	**Extraction solvent:**	90/10 MeOH/H_2O	**Crude extract:**	5.77 g
	**Column**	**Solvents**	**Gradient**	**Yield**
	SNAP KP-Sil 100 g	A: Petroleum ether<br>B: Ethyl acetate	10-100% (10 CV)<br>100% (3 CV)	140 mg
	Sfär C18 D 12 g	A: Water<br>B: Acetonitrile	60-100% (11 CV)	22 mg
	SNAP Ultra 10 g	A: Petroleum ether<br>B: Ethyl acetate	30% (2 CV)<br>30-67% (3 CV)<br>67% (5 CV)<br>67-100% (3 CV)<br>100% (5 CV)	12 mg

Table S3. ^{1}H and ^{13}C NMR data of 7,8-epoxymelianol (9) (C_6D_6, 298 K, 600 MHz).

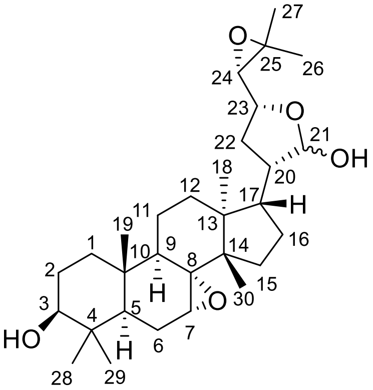

Atom	^{13}C ppm [a]	^{1}H ppm (m, Hz) [a]
1	38.05 / 37.98	0.77-0.83, 1.34-1.46 (m, 2H)
2	27.87	1.31-1.38 (m, 2H)
3	78.47 / 78.40	2.95-2.99 (m, 1H)
4	38.60	-
5	45.45 / 45.37	1.25-1.30 (m, 1H)
6	23.21 / 23.20	1.40-1.46, 1.91-1.96 (m, 2H)
7	55.05 / 54.96	2.70 (t, *J* = 1.7 Hz, 1H)
8	63.22 / 63.10	-
9	49.89 / 49.80[b]	1.85-1.91 (m, 1H)
10	35.08	-
11	17.65 / 17.61	1.42-1.48, 1.52-1.58 (m, 2H)
12	31.18 / 31.52	1.41-1.46, 1.98-2.04 (m, 2H) / 1.57-1.61, 1.78-1.93 (m, 2H)
13	45.34 / 45.61	-
14	49.09 / 49.38	-
15	28.29 / 27.84	0.82-0.87, 1.98-2.05 (m, 2H) / 0.80-0.82, 1.33-1.45 (m, 2H)
16	26.94 / 27.08	1.20-1.26, 1.71-1.81 (m, 2H)
17	45.89 / 51.25	2.17-2.20 (m, 1H) / 1.70-1.73 (m, 1H)
18	22.02 / 21.24	1.09 (s, 3H) /1.28 (s, 3H)
19	14.53 / 14.55	0.67 (s, 3H) / 0.70 (s, 3H)
20	47.19 / 49.72[b]	1.81-1.87 (m, 1H) / 2.17-2.21 (m, 1H)
21	97.87 / 102.2	5.29 (d, *J* = 3.9 Hz, 1H) / 5.31 (d, *J* = 3.4 Hz, 1H)
22	32.05 / 35.78	1.61-1.72 (m, 2H) / 1.15-1.20, 1.80-1.84 (m, 2H)
23	78.59 / 77.43	3.87-3.93 (m, 1H) / 4.02 (ddd, *J* = 10.7, 7.3, 5.1 Hz, 1H)
24	67.89 / 65.52	2.91 (d, *J* = 7.2 Hz, 1H) / 2.78 (d, *J* = 7.3 Hz, 1H)
25	57.41 / 56.41	-
26	25.17 / 25.02	1.14 (s, 3H) / 1.12 (s, 3H)
27	19.32 / 19.59	1.12 (s, 3H) /1.09 (s, 3H)
28	28.09 / 28.05	0.97 (s, 3H)
29	16.04 / 16.06	0.84 (s, 3H)
30	22.97 / 22.81	0.94 (s, 3H) / 0.90 (s, 3H)

[a] Second value corresponds to the minor lactol epimer whenever clearly discernible.

[b] Signals interchangeable.

Table S4. **^1H and ^{13}C NMR data of isomeliandiol (10) obtained as a mixture of epimers ($CDCl_3$, 298 K, 500 MHz).**

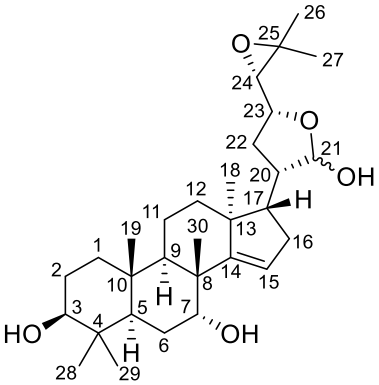

Atom	^{13}C ppm [a]	^1H ppm (m, Hz) [a]
1	38.05 / 38.03	1.03-1.07, 1.57-1.63 (m, 2H)
2	27.29	1.55-1.66 (m, 2H)
3	78.92 / 78.89	3.27 (dd, *J* = 11.1 Hz, 1H) / 3.28 (dd, *J* = 4.7 Hz, 1H)
4	38.51	-
5	46.68 / 46.64	1.45-1.52 (m, 1H)
6	23.81 / 23.83	1.70-1.78, 1.82-1.88 (m, 2H)
7	72.47 / 72.48	3.92 (m, 1H)
8	44.39 / 44.38	-
9	41.88 / 41.93	1.88-1.94 (m, 1H)
10	37.73	-
11	16.48 / 16.47	1.48-1.53, 1.67-1.72 (m, 2H)
12	33.22 / 33.27	1.49-1.53, 1.77-1.81 (m, 2H)
13	46.85 / 46.78	-
14	162.35 / 162.22	-
15	119.67 / 119.18	5.47 (m, 1H) / 5.45 (m, 1H)
16	35.22 / 35.14	2.15-2.20 (m, 2H)
17	52.88 / 52.96	1.92-2.01 (m, 1H)
18	19.94 / 19.96	1.02 (s, 3H)
19	15.54	0.89 (s, 3H)
20	45.63 / 47.95	1.46-1.51 (m, 1H)
21	97.74 / 102.55	5.38 (d, *J* = 3.2 Hz, 1H)
22	31.50 / 34.87	1.69-1.77, 2.00-2.05 / 1.36-1.44, 2.07-2.15 (m, 2H)
23	78.53 / 77.36	3.88-3.94 (m, 1H) / 3.93-3.99 (m, 1H)
24	67.77 / 65.35	2.82 (d, *J* = 7.4 Hz, 1H) / 2.69 (d, *J* = 7.6 Hz, 1H)
25	58.27 / 57.46	-
26	25.17 / 25.06	1.32 (s, 3H) / 1.33 (s, 3H)
27	19.35 / 19.59	1.32 (s, 3H) / 1.09 (s, 3H)
28	27.81	0.99 (s, 3H)
29	15.59	0.79 (s, 3H)
30	27.80	1.05 (s, 3H)

[a] Second value corresponds to the minor lactol epimer whenever clearly discernible.

Table S5. Partial ^{1}H and ^{13}C NMR data of degradation products 12 and 13 present in NMR samples of isomeliandiol (10) ($CDCl_3$, 298 K, 500 MHz).

	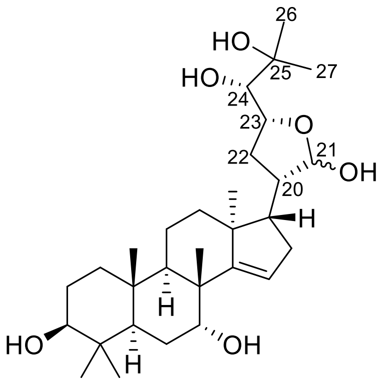 **12**		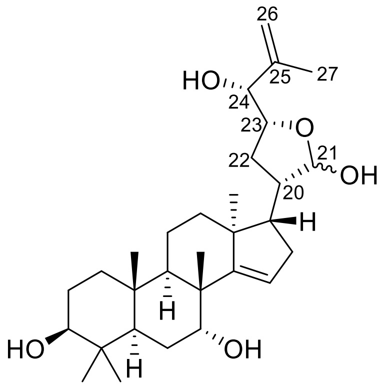 **13**	
**Atom**	**^{13}C ppm**	**^{1}H ppm**	**^{13}C ppm**	**^{1}H ppm**
20	45.13	2.17	45.79	2.22
21	97.54	5.33	97.30	5.29
22	30.19	1.95	30.52	1.81
23	78.69	4.46	80.31	4.23
24	75.37	3.19	77.23	3.90
25	73.35	-	145.09	-
26	26.77	1.29	112.62	4.94, 5.04
27	26.91	1.27	18.87	1.78

Table S6. ^{1}H and ^{13}C NMR data of protoglabretal (11) ($CDCl_3$, 298 K, 500 MHz).

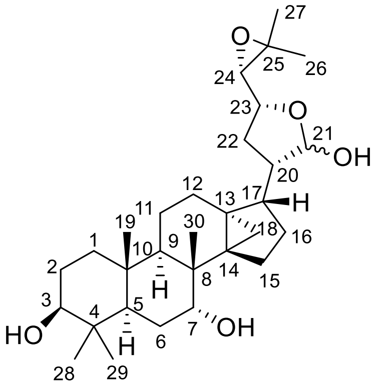

Atom	^{13}C ppm [a]	^{1}H ppm (m, Hz) [a]
1	38.70 / 38.58	0.92-1.00, 1.57-1.63 (m, 2H)
2	27.28	1.59-1.67 (m, 2H)
3	78.92 / 78.89	3.26 (dd, *J* = 11.4, 4.3 Hz, 1H)
4	38.47 / 38.46	-
5	46.11 / 46.03	1.43-1.48 (m, 1H)
6	24.35 / 34.34	1.56-1.63, 1.71-1.77 (m, 2H)
7	74.65 / 74.51	3.74-3.79 (m, 1H)
8	38.90 / 38.97	-
9	44.32 / 44.12	1.16-1.24 (m, 1H)
10	37.34 / 37.40	-
11	25.75	1.70-1.78, 2.06-2.14 (m, 2H)
12	16.46 / 16.30	1.23-1.36 (m, 2H)
13	29.07 / 28.72	-
14	37.03 / 36.23	-
15	26.45 / 27.26	1.88-1.95 (m, 2H) / 1.49-1.58 (m, 2H)
16	27.63 / 26.29	0.85-0.88, 1.62-1.68 (m, 2H) / 0.89-0.93, 1.62-1.68 (m, 2H)
17	44.90 / 48.44	2.15-2.24 (m, 1H) / 1.99-2.05 (m, 1H)
18	13.88 / 13.65	0.45 (d, *J* = 4.9 Hz, 1H), 0.65 (d, *J* = 4.8 Hz, 1H) / 0.47 (d, *J* = 5.3 Hz, 1H), 0.73 (d, *J* = 5.1 Hz, 1H)
19	16.06 / 15.93	0.87 (s, 3H)
20	49.48 / 50.91	1.82-1.90 (m, 1H) / 2.10-2.15 (m, 1H)
21	98.34 / 102.25	5.42 (d, *J* = 3.9 Hz, 1H) / 5.42 (d, *J* = 3.4 Hz, 1H)
22	30.93 / 33.13	1.65-1.74, 1.94-1.99 (m, 2H) / 1.37-1.43, 2.02-2.08 (m, 2H)
23	78.57 / 77.46	3.84-3.91 (m, 1H) /3.92-3.97 (m, 1H)
24	67.78 / 65.41	2.83 (d, *J* = 7.5 Hz, 1H) /2.69 (d, *J* = 7.6 Hz, 1H)
25	58.31 / 57.51	-
26	25.14 / 25.05	1.31 (s, 3H) / 1.33 (s, 3H)
27	19.32 / 19.56	1.30 (s, 3H) / 1.02 (s, 3H)
28	27.87 / 27.86	0.97 (s, 3H)
29	15.68 / 15.67	0.77 (s, 3H)
30	19.66 / 19.53	1.03 (s, 3H)

[a] Second value corresponds to the minor lactol epimer whenever clearly discernible.

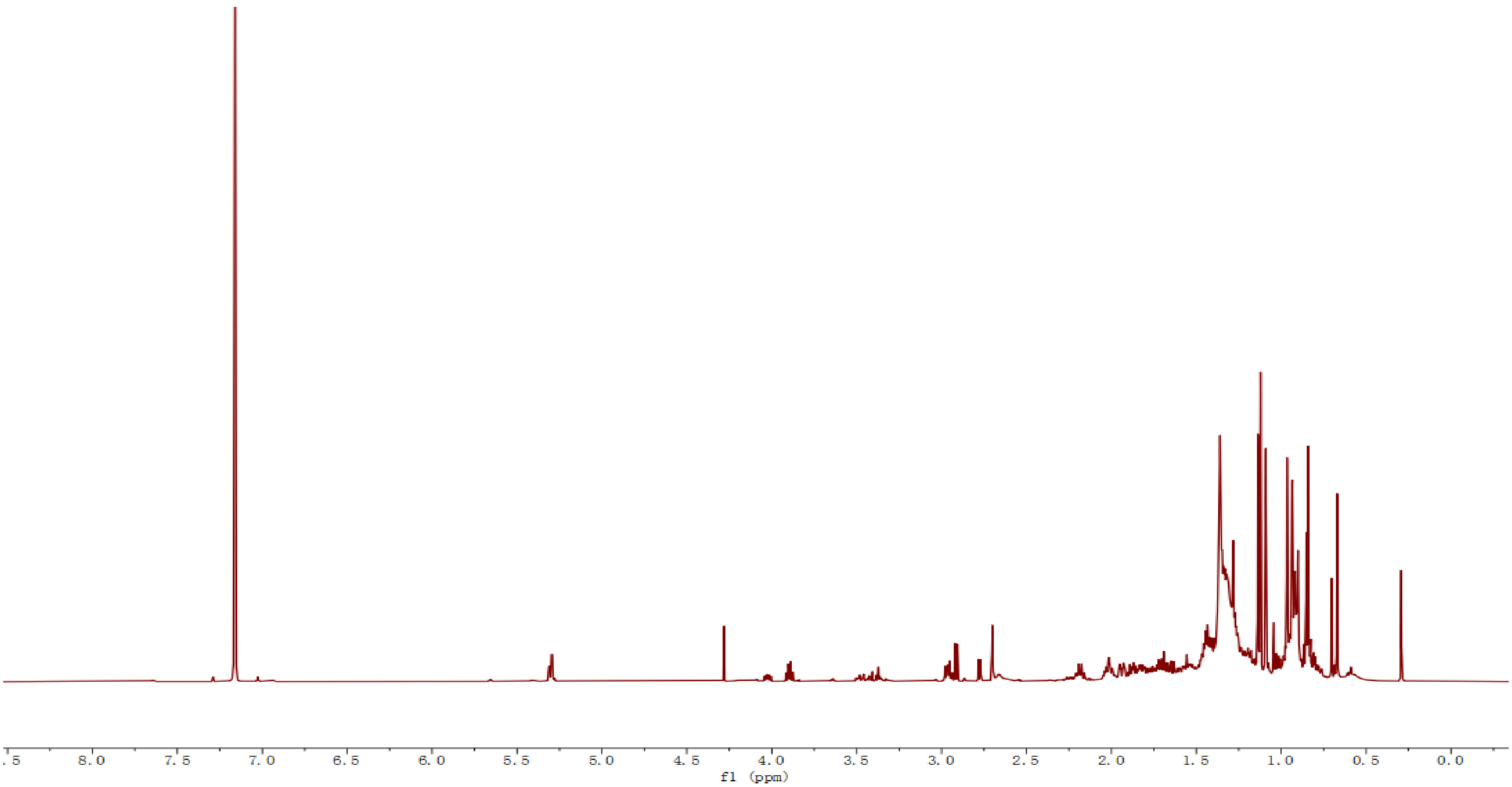

Figure S5. ^{1}H spectrum of 7,8-epoxymelianol (9) (C_6D_6, 298 K, 600 MHz).

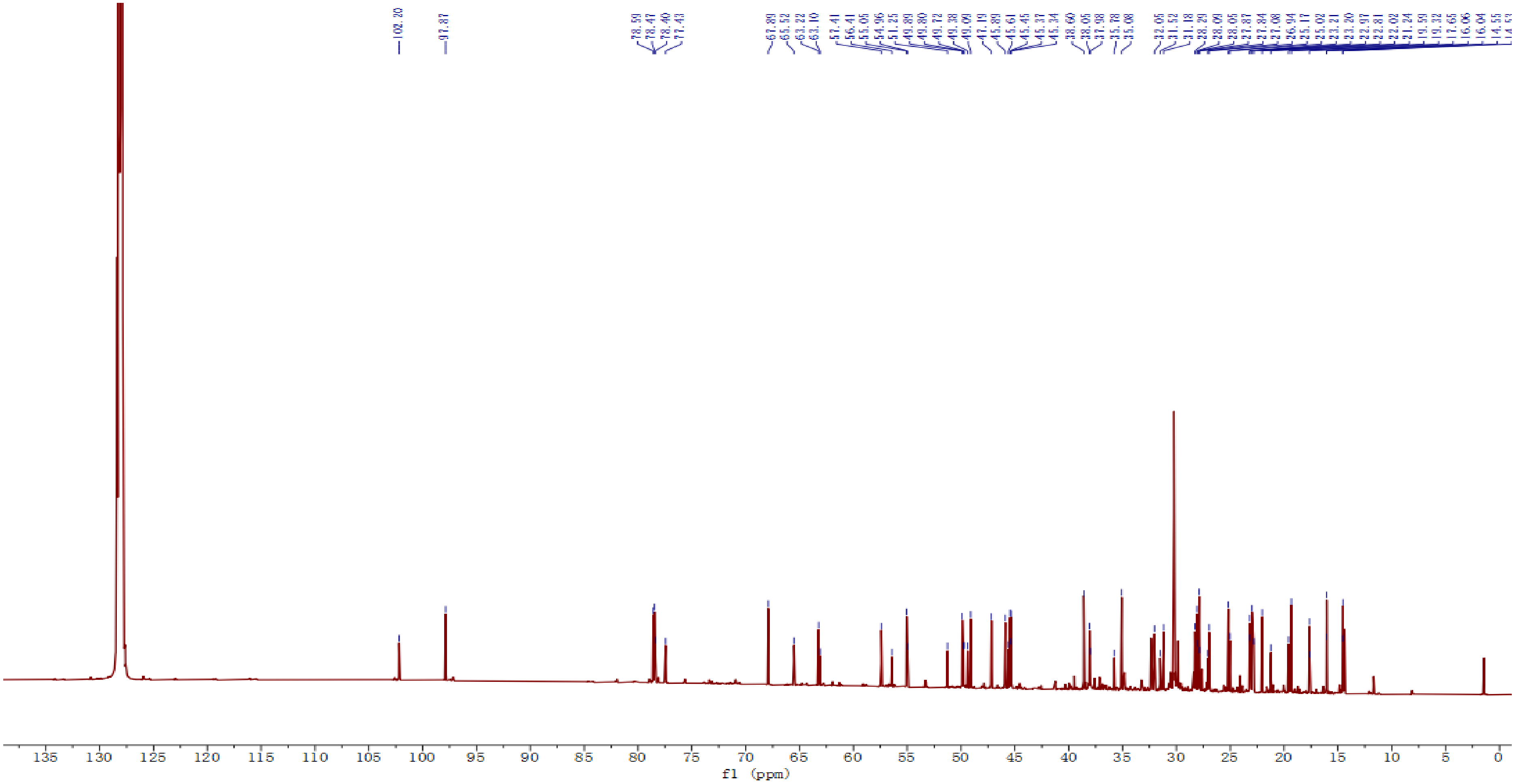

Figure S6. ^{13}C **spectrum of 7,8-epoxymelianol (9) (C_6D_6, 298 K, 151 MHz).**

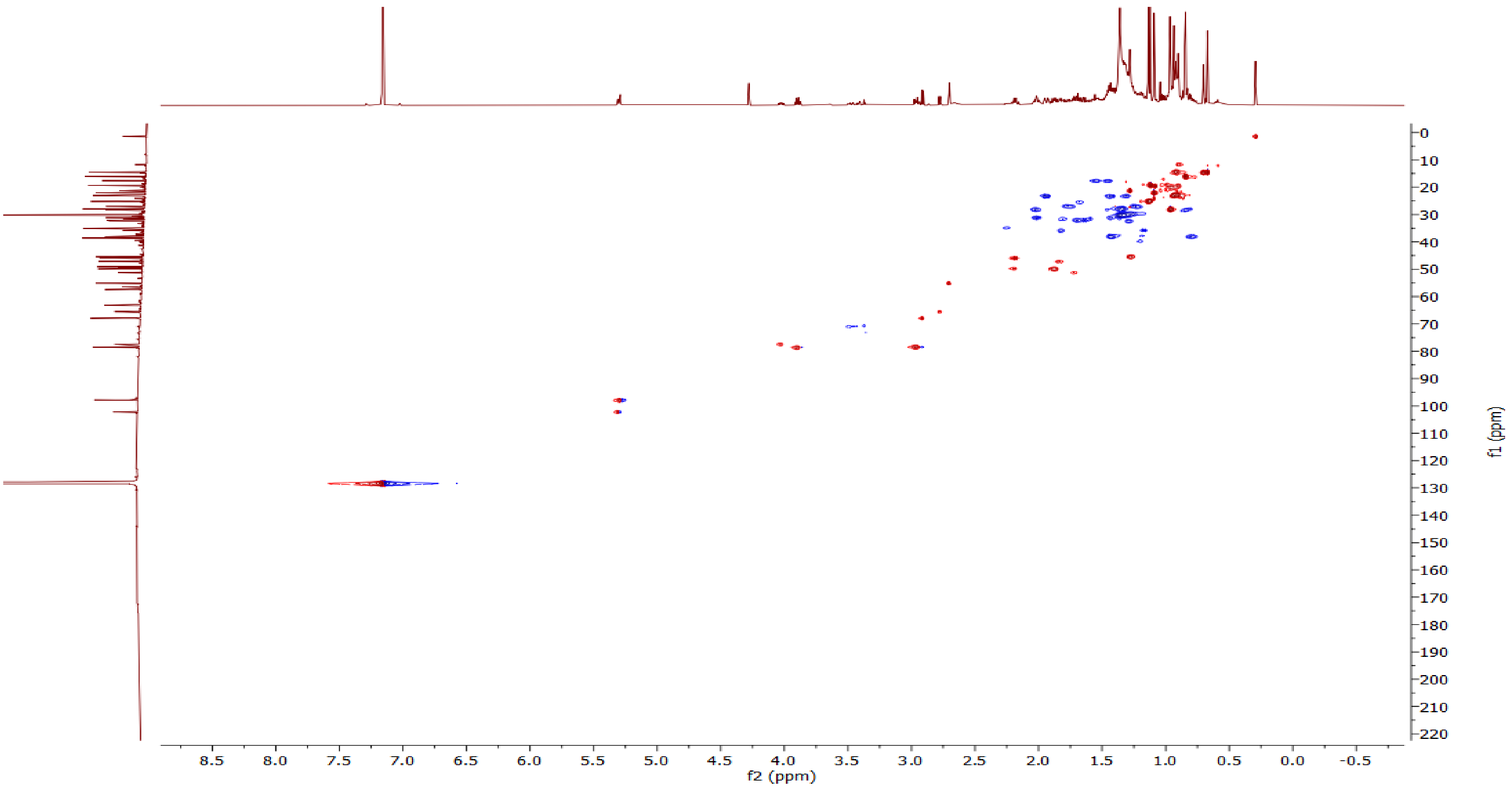

Figure S7. HSQC spectrum of 7,8-epoxymelianol (9) (C_6D_6, 298 K, 600 MHz).

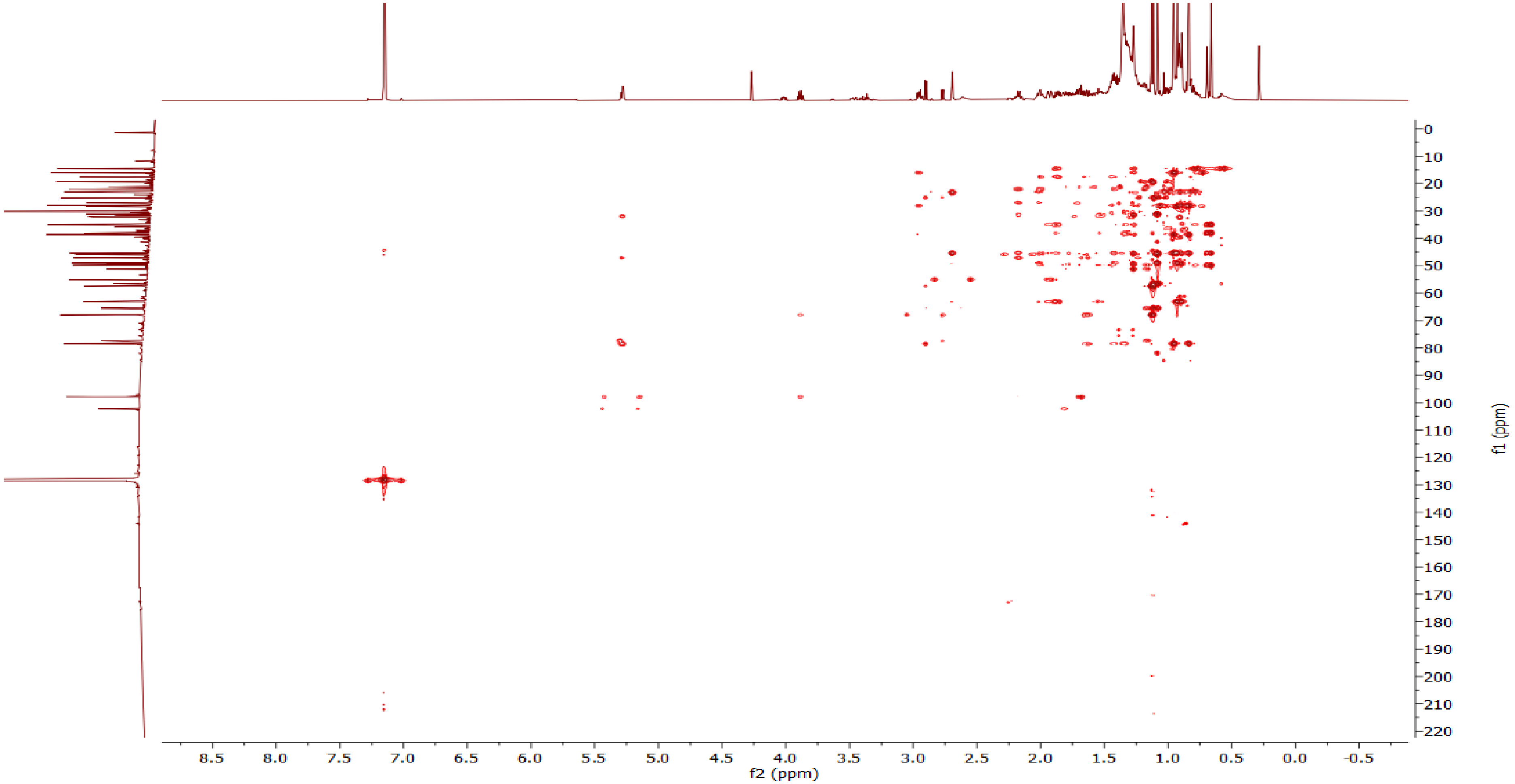

Figure S8. HMBC spectrum of 7,8-epoxymelianol (9) (C_6D_6, 298 K, 600 MHz).

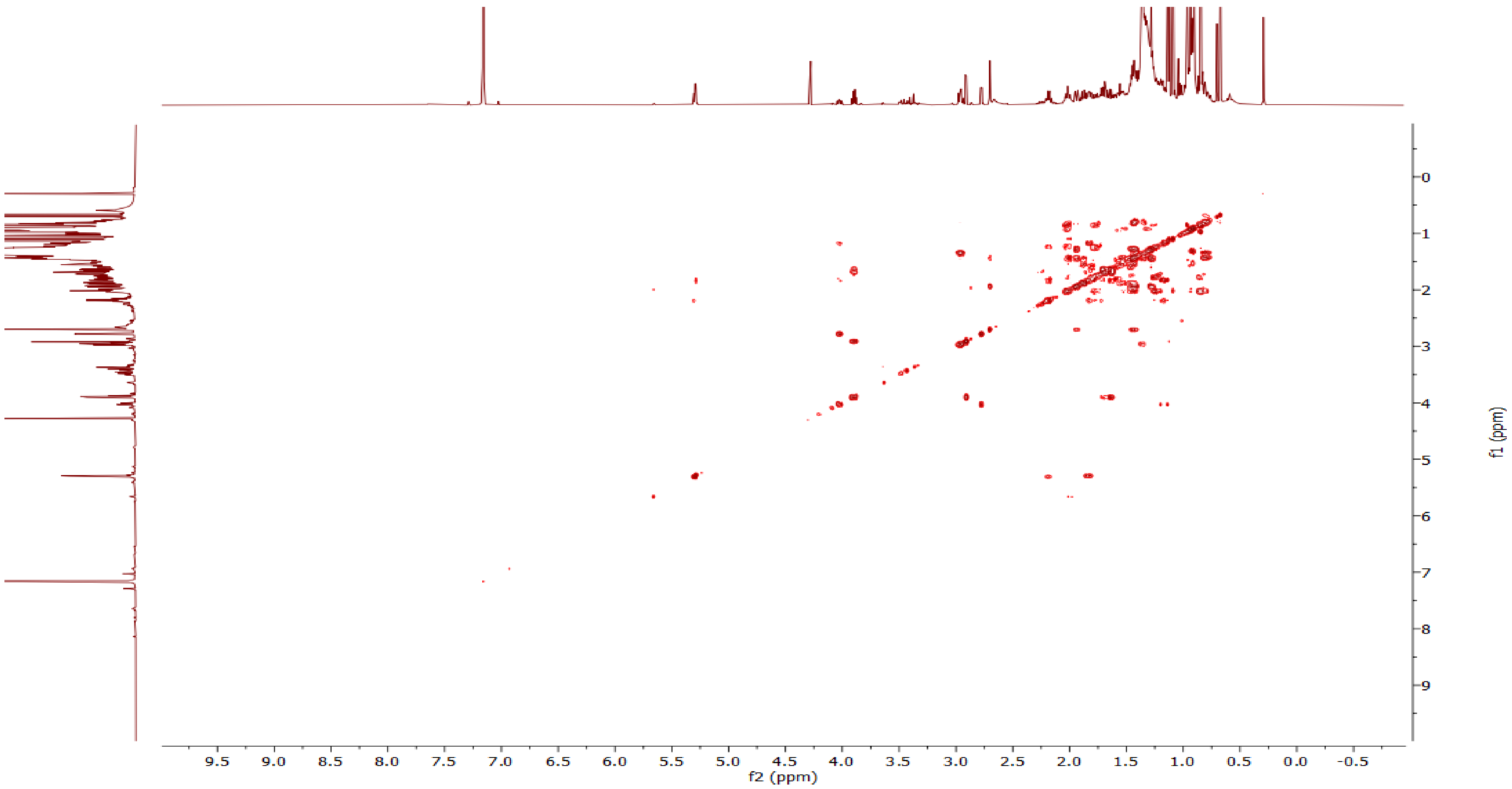

Figure S9. COSY spectrum of 7,8-epoxymelianol (9) (C_6D_6, 298 K, 600 MHz).

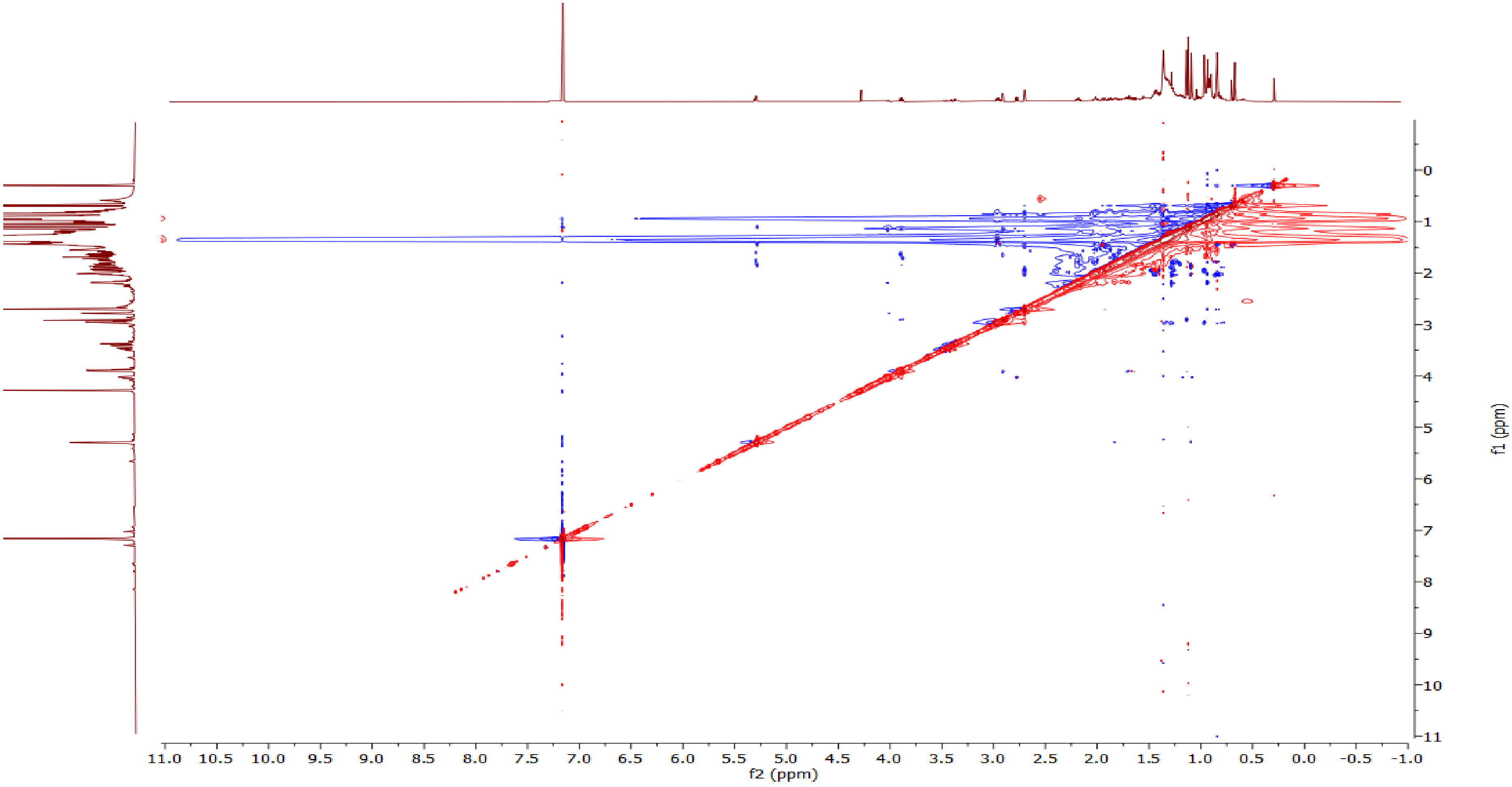

Figure S10. NOESY spectrum of 7,8-epoxymelianol (9) (C_6D_6, 298 K, 500 MHz).

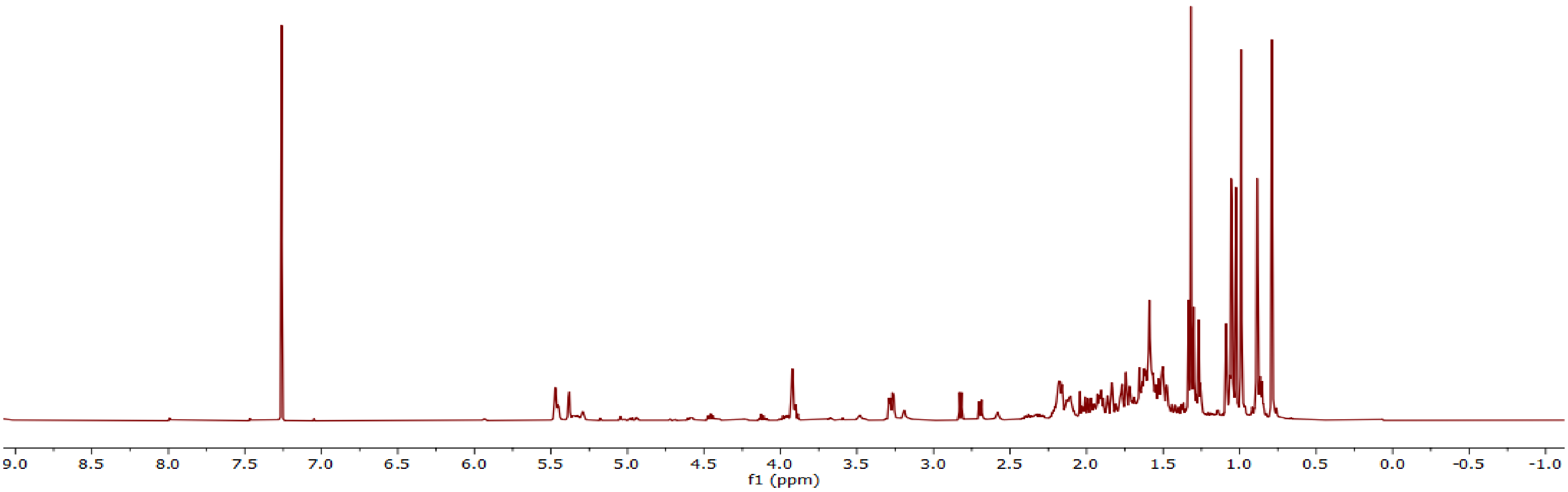

Figure S11. ^{1}H NMR spectrum of isomeliandiol (10) ($CDCl_3$, 298 K, 500 MHz).

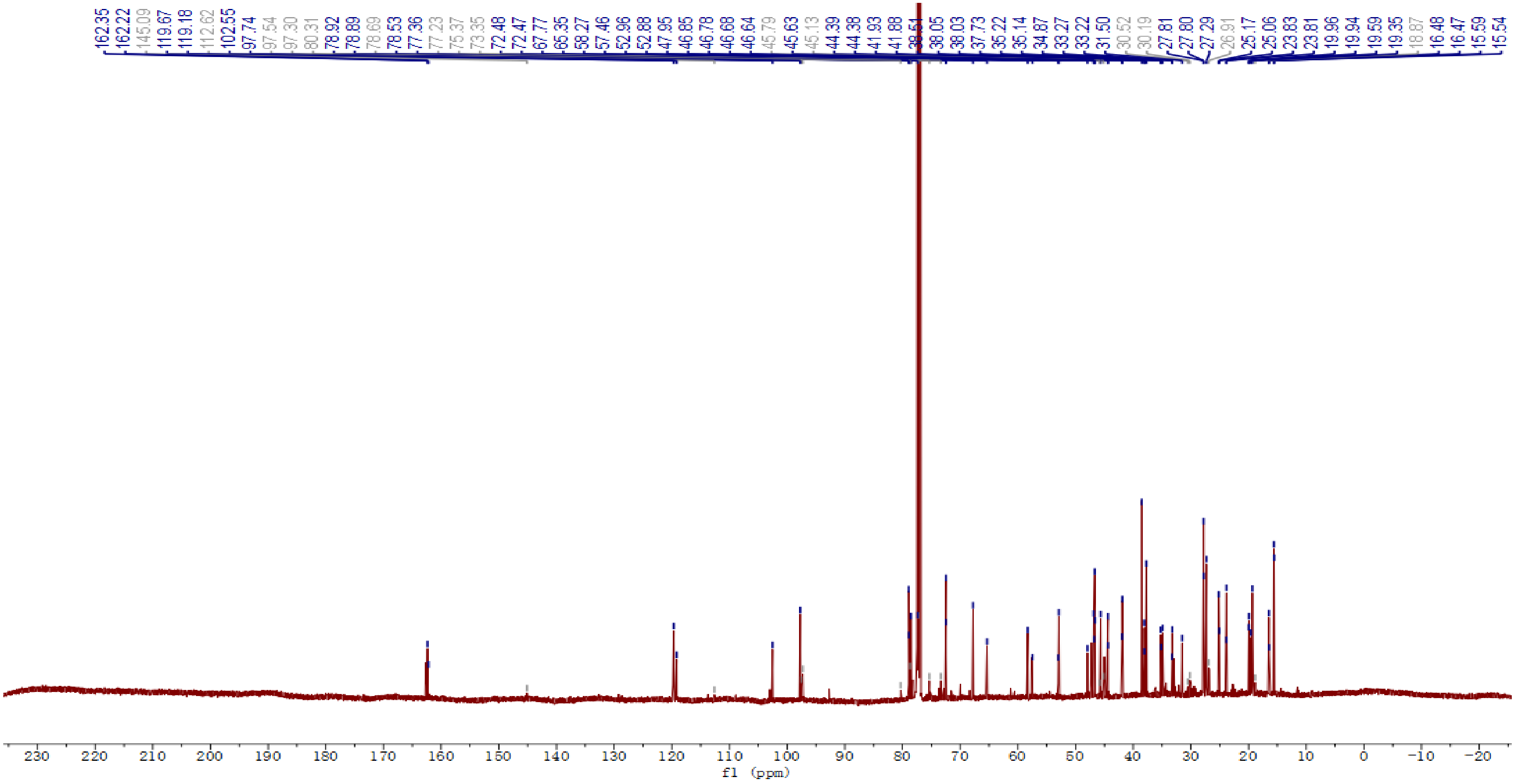

Figure S12. ^{13}C NMR spectrum of isomeliandiol (10) containing the C24/25 epoxide degradation products 12 and 13 ($CDCl_3$, 298 K, 126 MHz). The ^{13}C resonances of isomeliandiol (**10**) are shown in blue and key resonances of degradation products **12** and **13** are shown in grey.

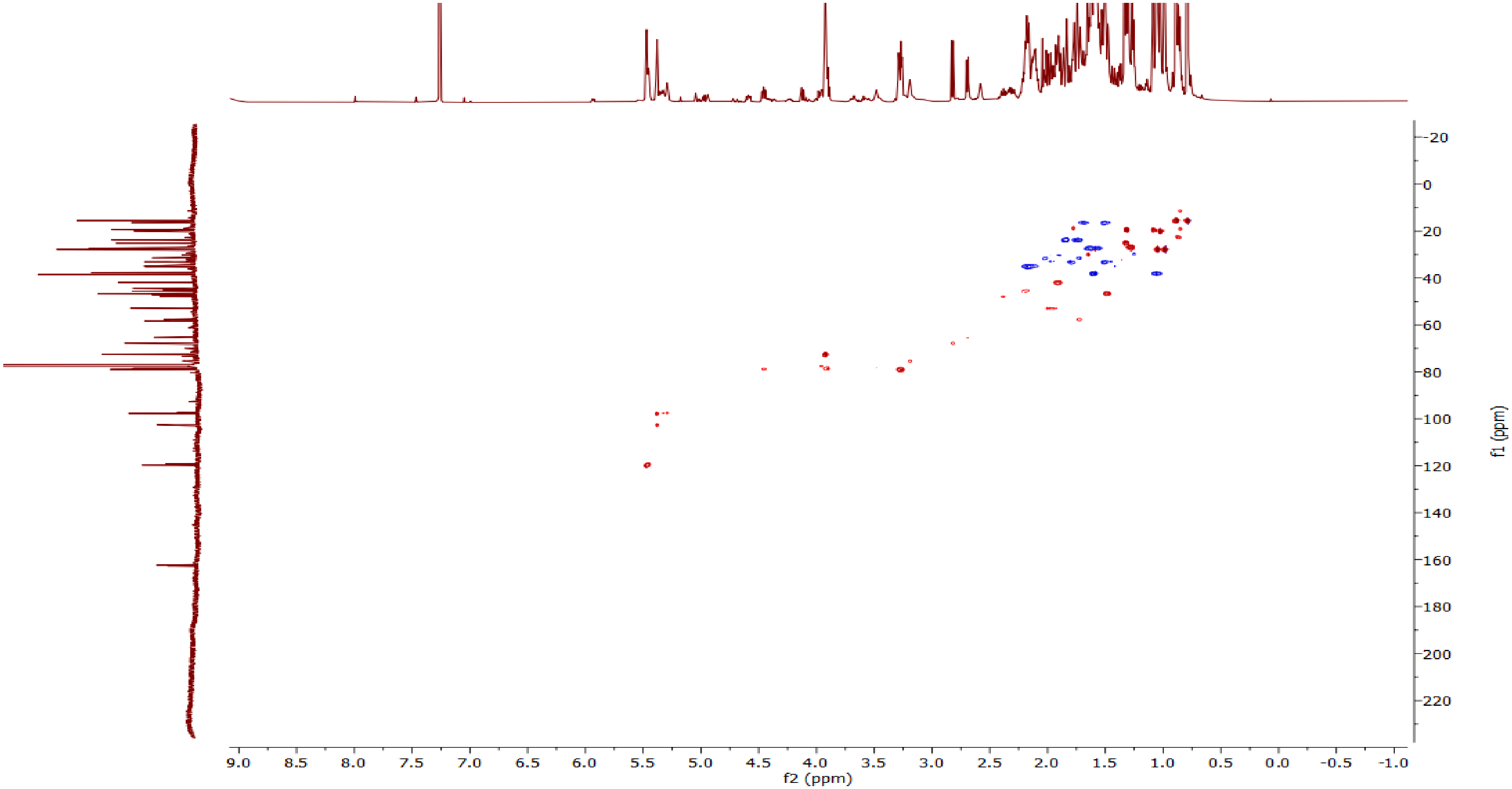

Figure S13. HSQC spectrum of isomeliandiol (10) ($CDCl_3$, 298 K, 500 MHz).

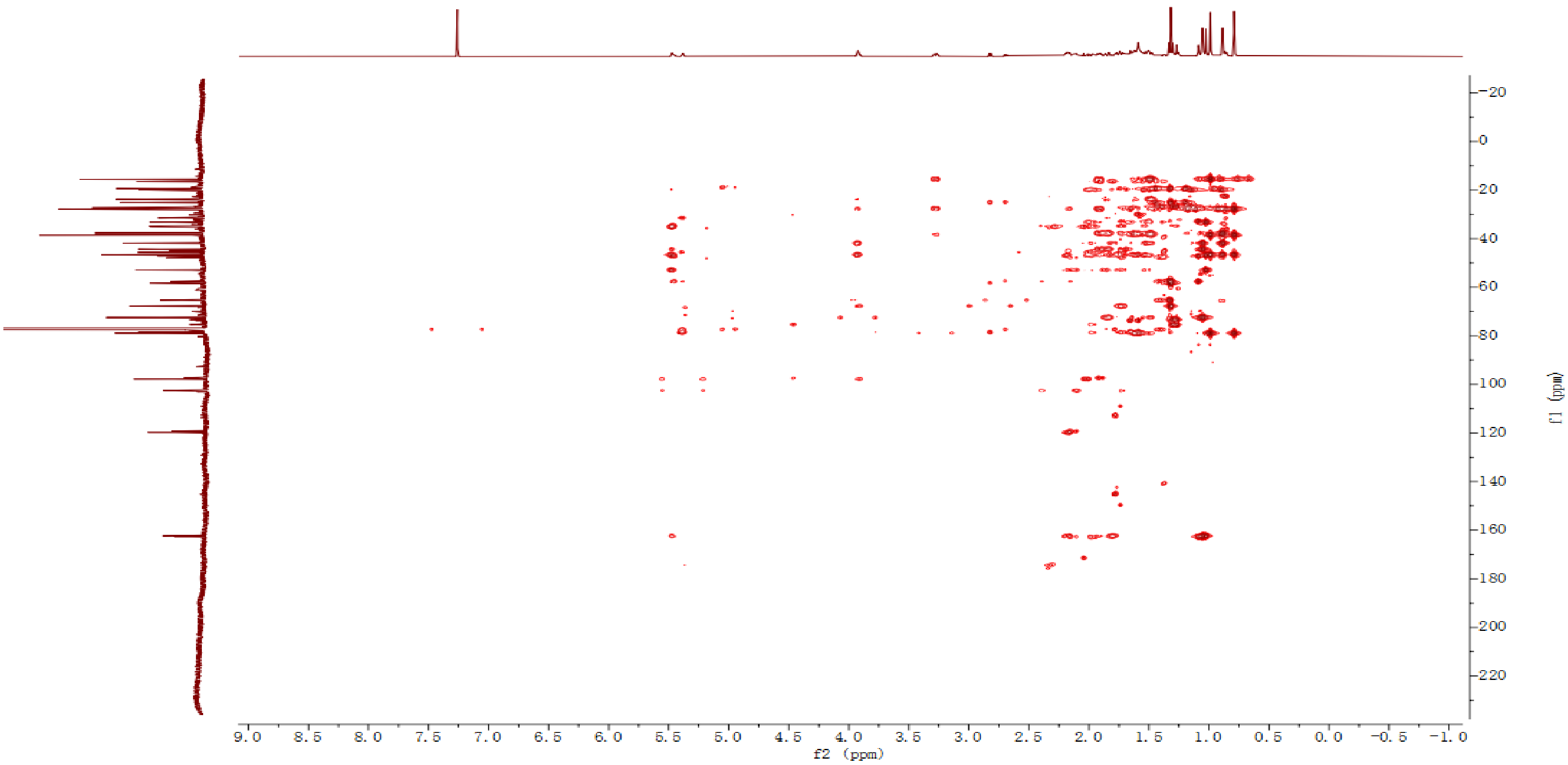

Figure S14. HMBC spectrum of isomeliandiol (10) ($CDCl_3$, 298 K, 500 MHz).

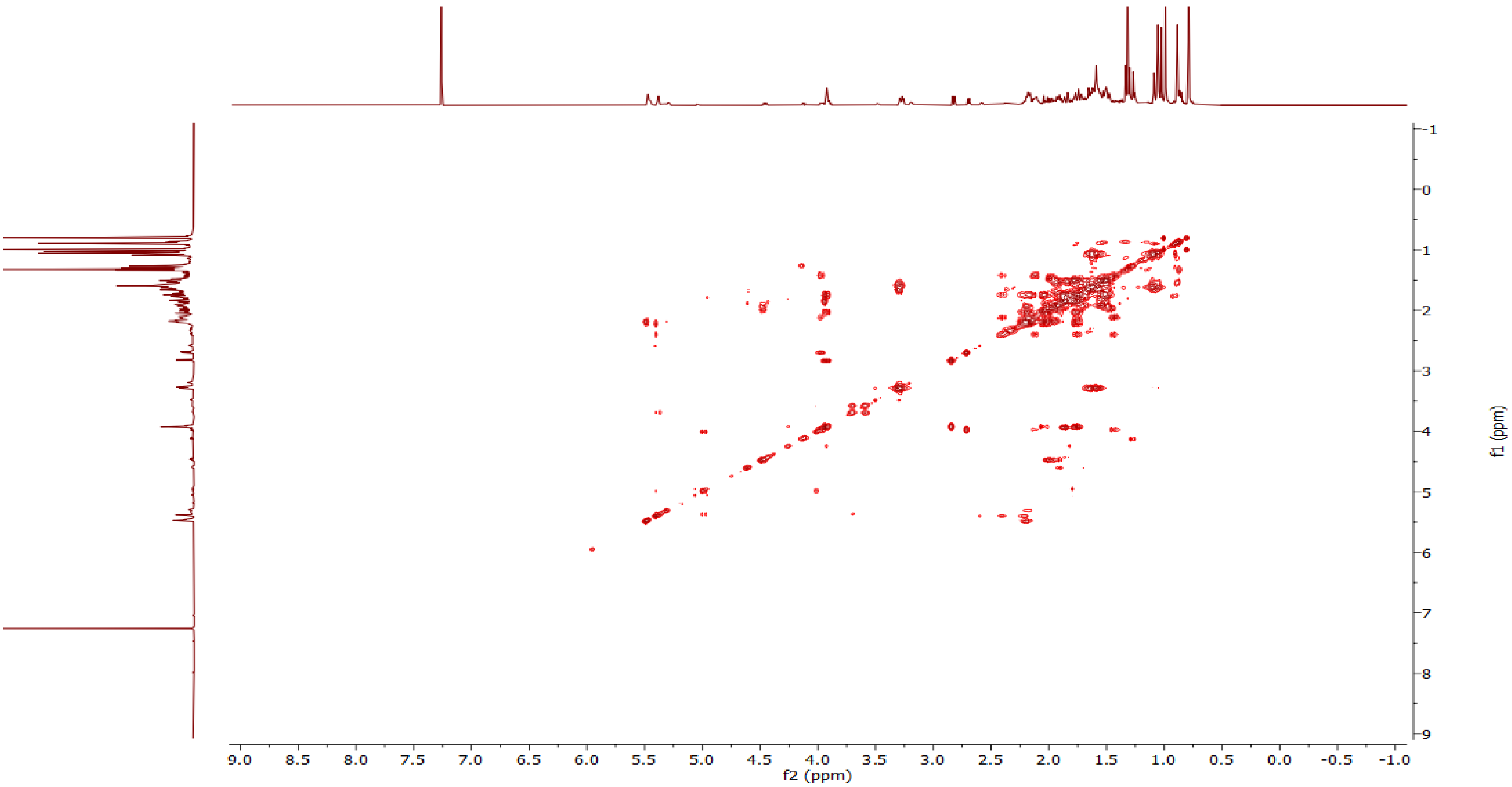

Figure S15. COSY spectrum of isomeliandiol (10) ($CDCl_3$, 298 K, 500 MHz).

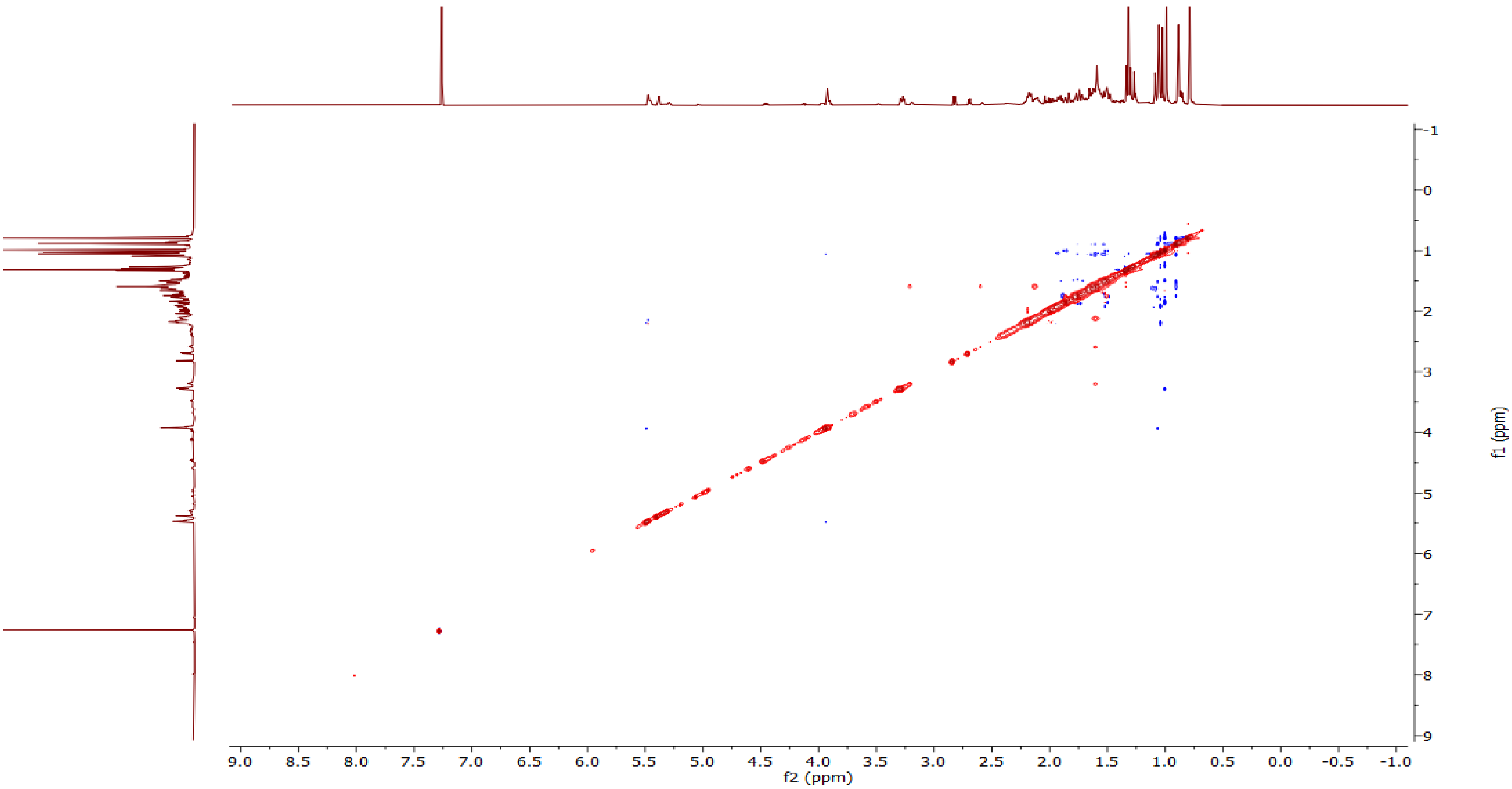

Figure S16. NOESY spectrum of isomeliandiol (10) ($CDCl_3$, 298 K, 500 MHz).

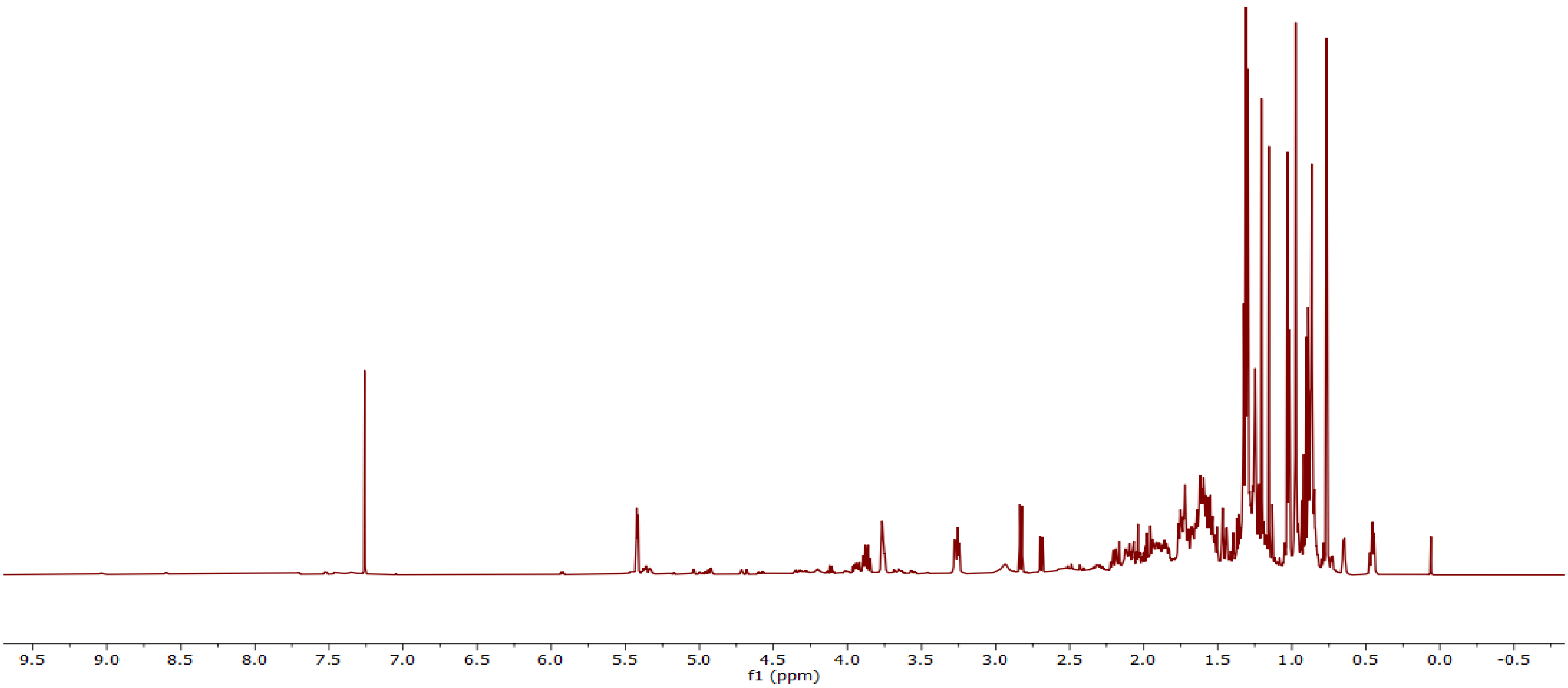

Figure S17. ^{1}**H NMR spectrum of protoglabretal (11) (CDCl$_3$, 298 K, 500 MHz).**

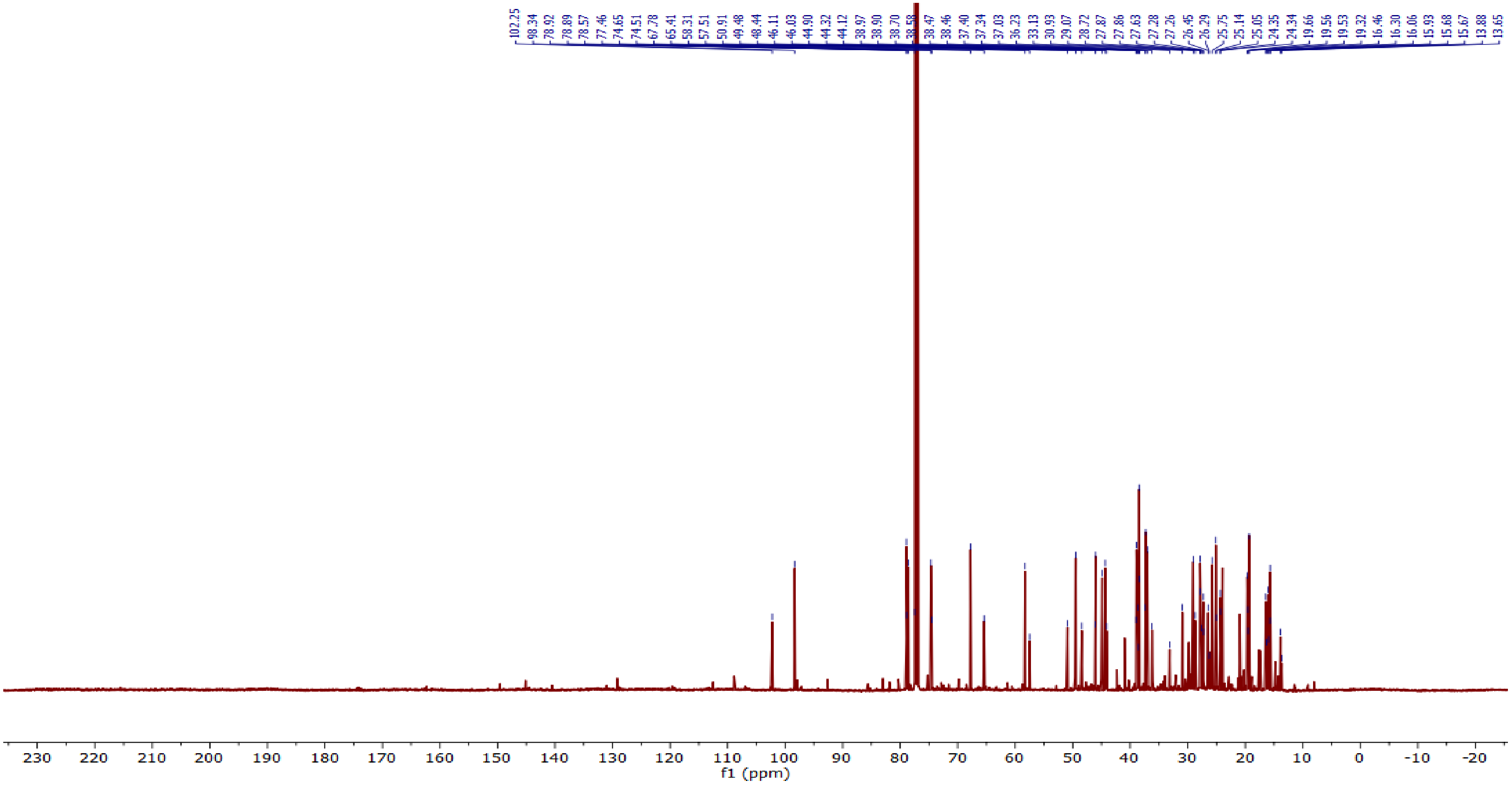

Figure S18. ^{13}C **NMR spectrum of protoglabretal (11) (**$CDCl_3$**, 298 K, 126 MHz).**

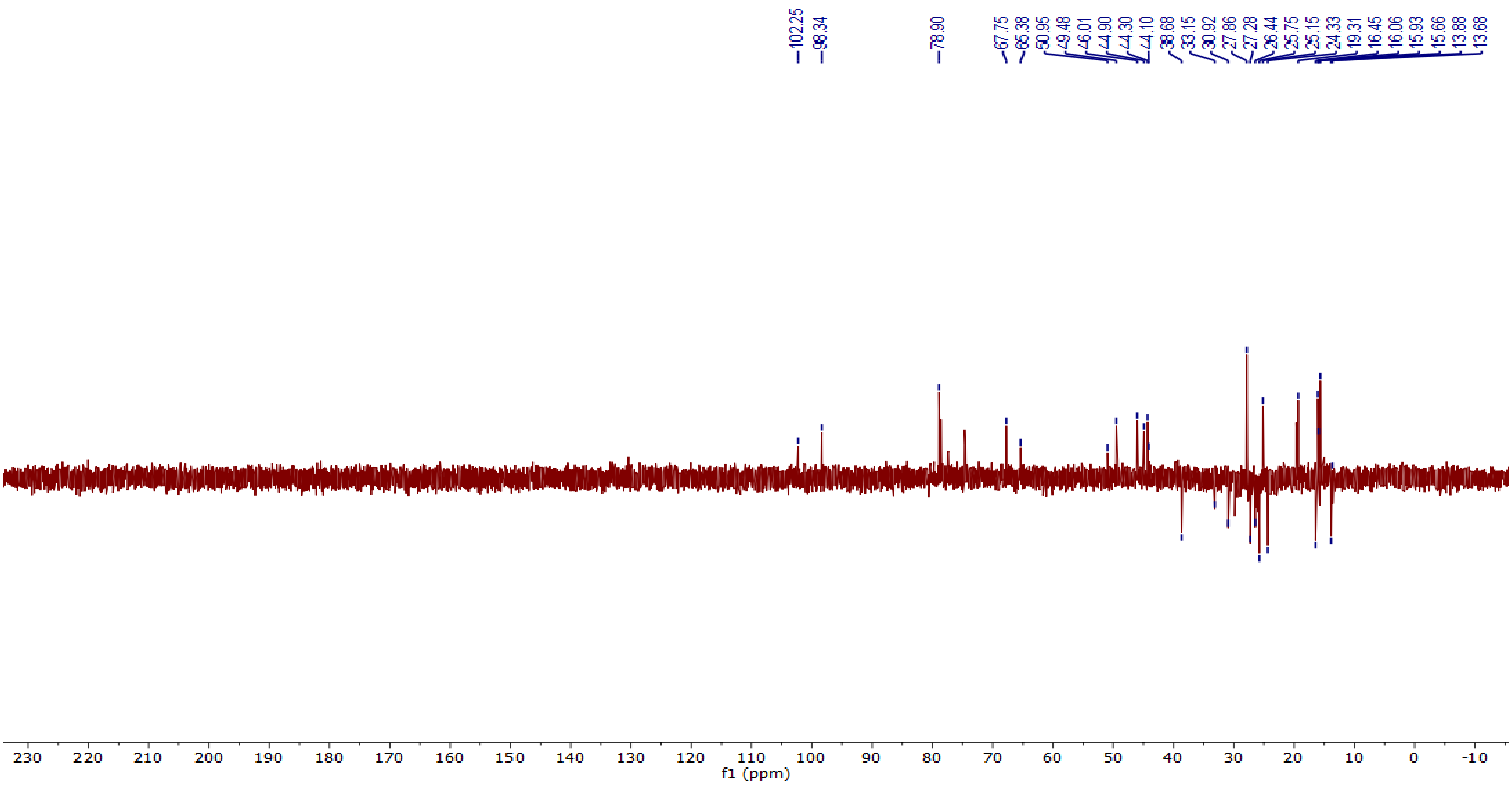

Figure S19. DEPT-135 spectrum of protoglabretal (11) ($CDCl_3$, 298 K, 100 MHz).

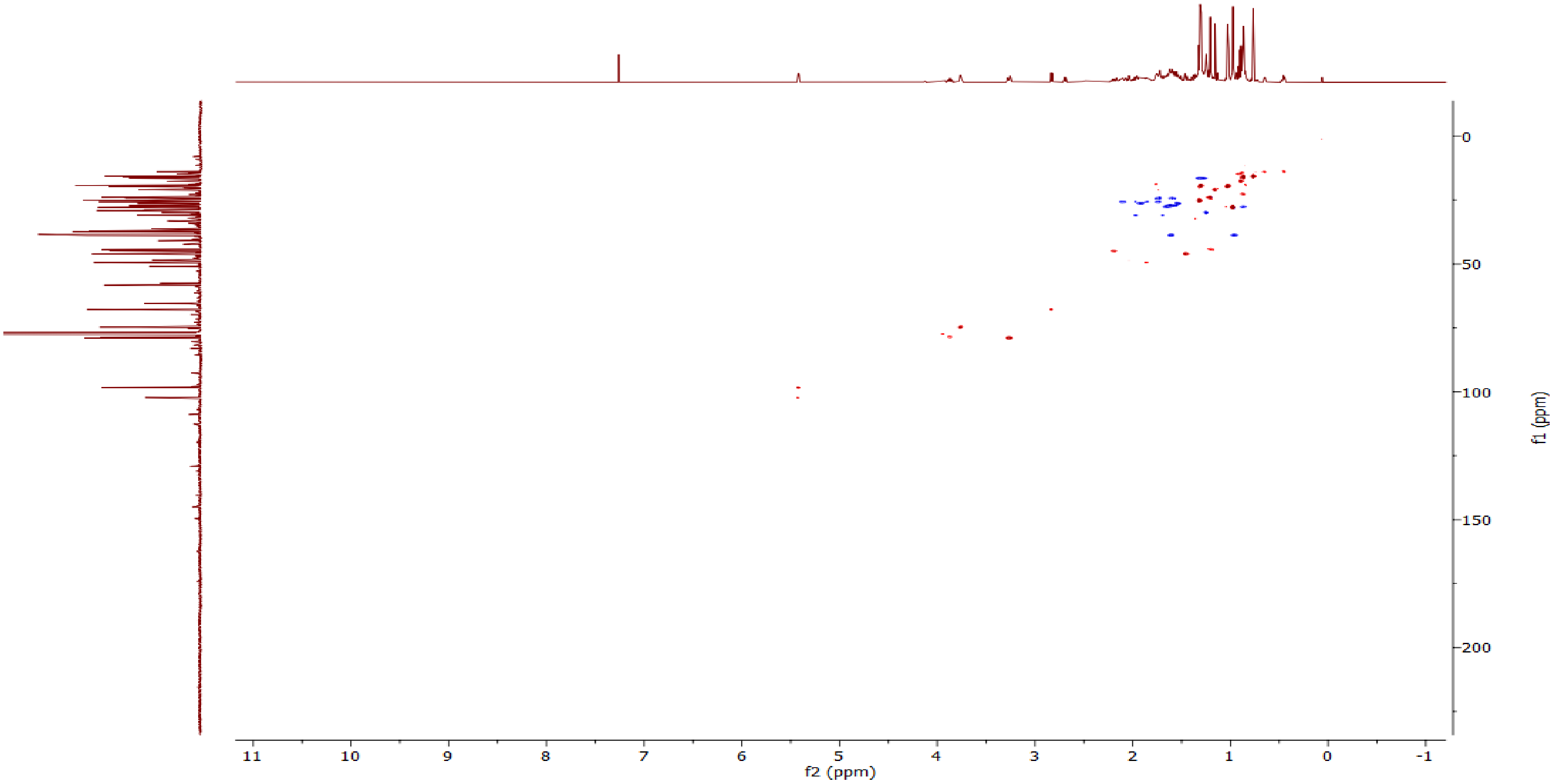

Figure S20. HSQC spectrum of protoglabretal (11) ($CDCl_3$, 298 K, 500 MHz).

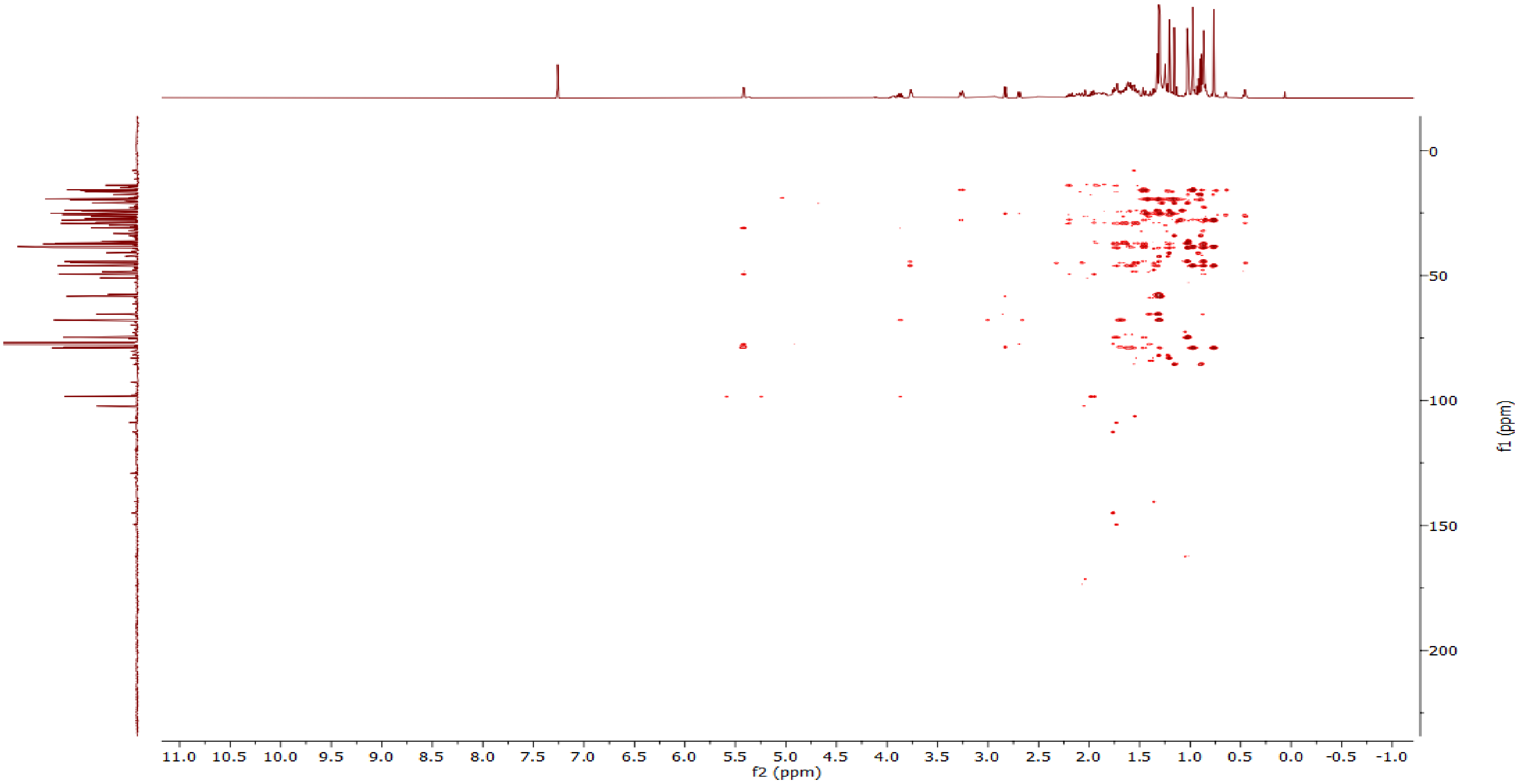

Figure S21. HMBC spectrum of protoglabretal (11) ($CDCl_3$, 298 K, 500 MHz).

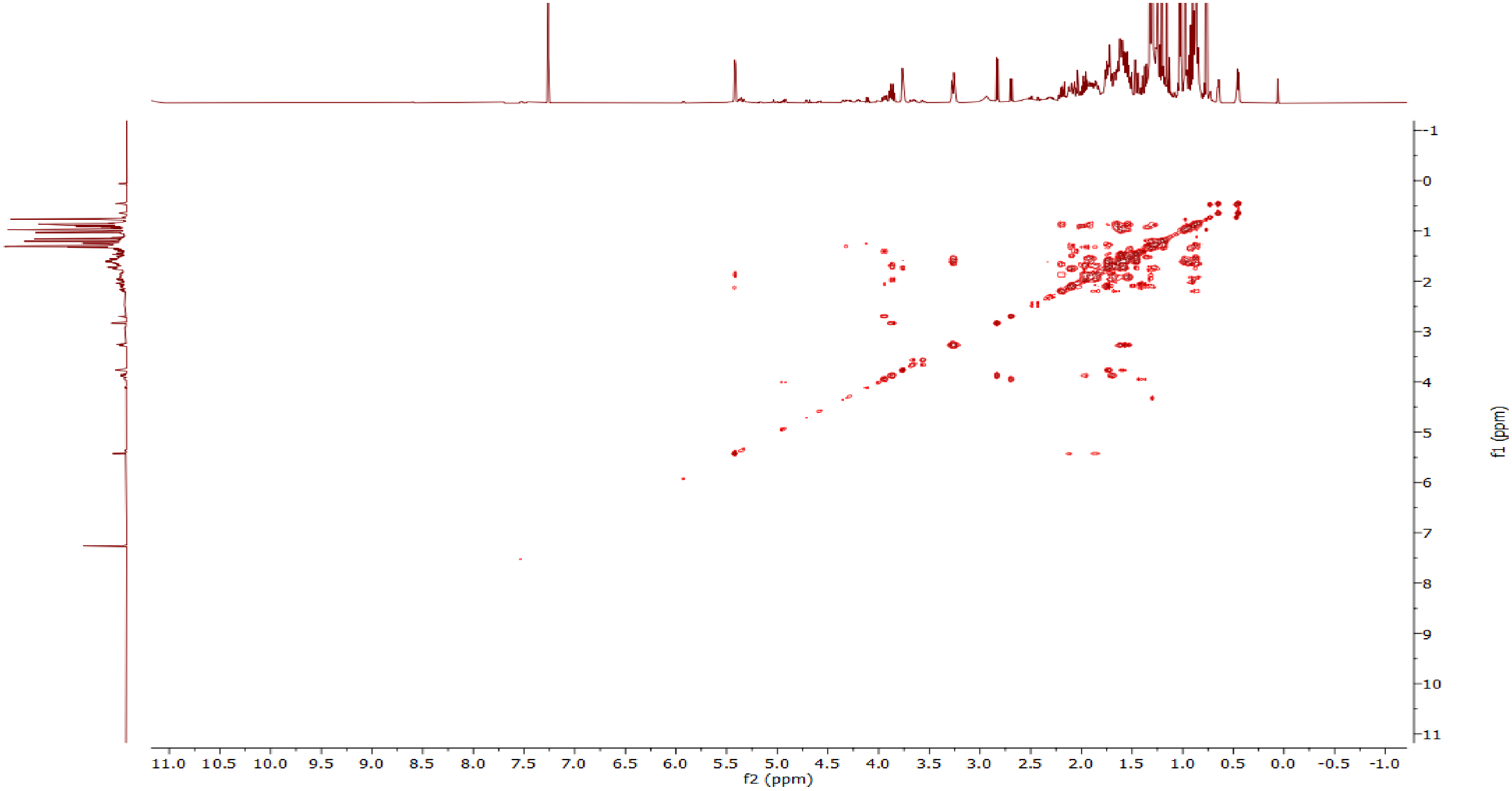

Figure S22. COSY spectrum of protoglabretal (11) ($CDCl_3$, 298 K, 500 MHz).

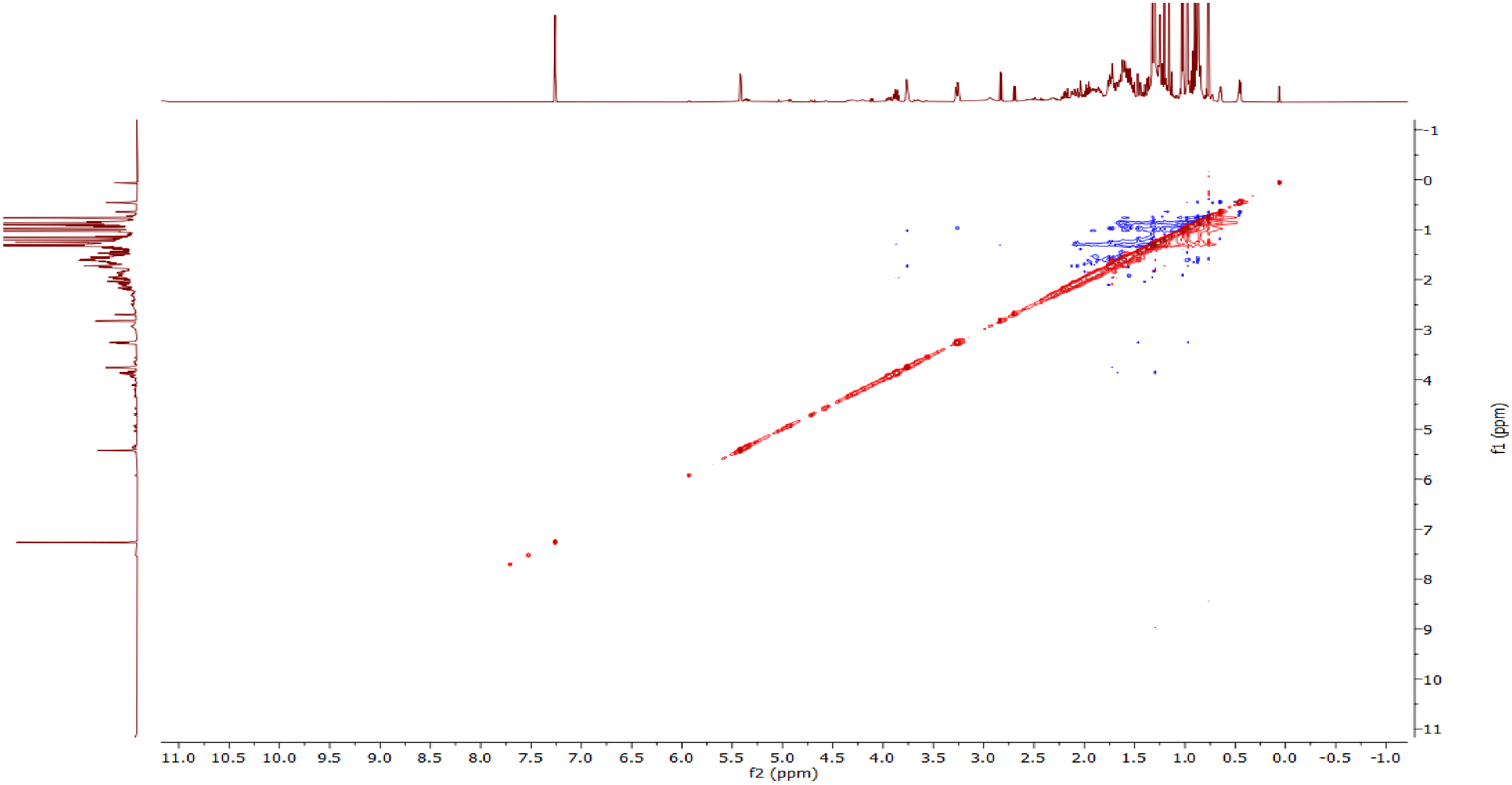

Figure S23. NOESY spectrum of protoglabretal (11) ($CDCl_3$, 298 K, 500 MHz).

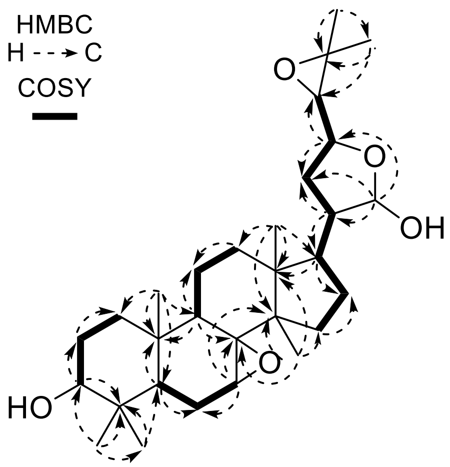

Figure S24. Selected key HMBC and COSY correlations of 7,8-epoxymelianol (9).

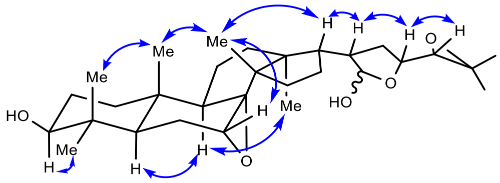

Figure S25. Selected NOE correlations of 7,8-epoxymelianol (9).

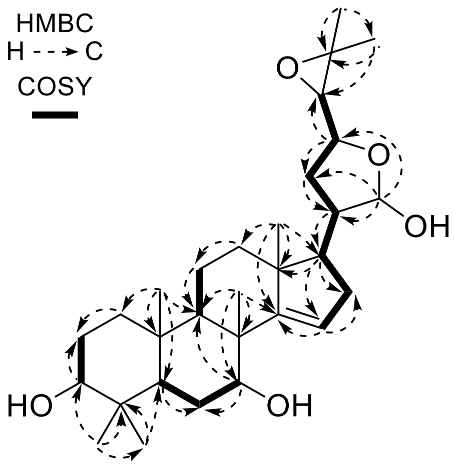

Figure S26. Selected key HMBC and COSY correlations of isomeliandiol (10).

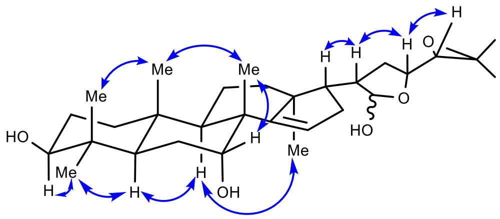

Figure S27. Selected NOE correlations of isomeliandiol (10).

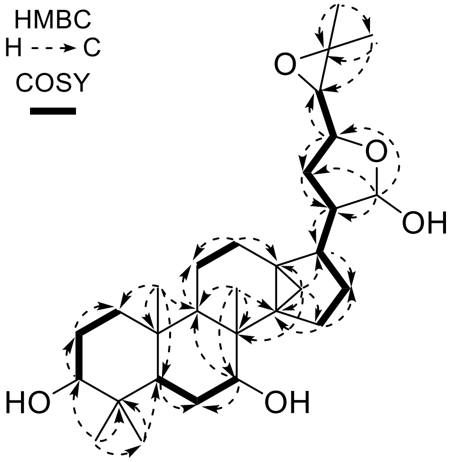

Figure S28. Selected key HMBC and COSY correlations of protoglabretal (11).

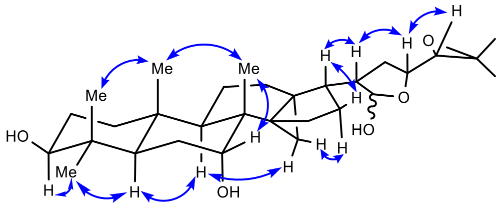

Figure S29. Selected NOE correlations of protoglabretal (11).

Table S7. Representative examples of apoprotolimonoids previously isolated from Meliaceae, Rutaceae, and Simaroubaceae plants out of ca. 120 structurally related natural products listed in Reaxys. Stereochemistry is shown as reported in the literature.

Trivial name	Structure	Family	Subfamily	Species	Reference
No trivial name	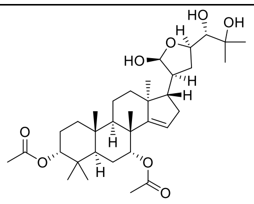	Meliaceae	Aglaieae	*Aglaia odorata* var. *microphyllina*	35
Agladoral C	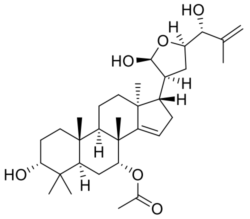	Meliaceae	Aglaieae	*Aglaia odorata* var. *microphyllina*	35
Toonasinensin A	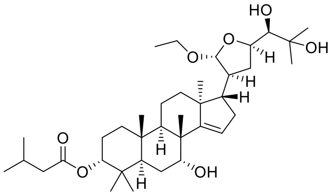	Meliaceae	Cedreleae	*Toona sinensis*	36
No trivial name	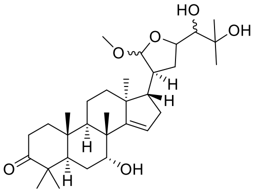	Meliaceae	Cedreleae	*Cedrela sinensis*	37
Toonaciliatine A	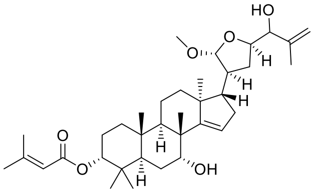	Meliaceae	Cedreleae	*Toona ciliata*	38
21-*O*-Acetyl-toosendantriol	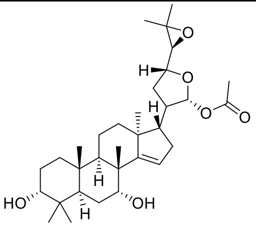	Meliaceae	Melioideae	*Melia toosendan*	39
Cumingianol D	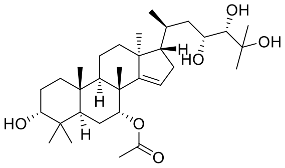	Meliaceae	Melioideae	*Dysoxylum cumingianum*	40

Lepidotrichilin B	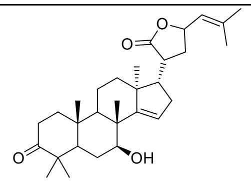	Meliaceae	Trichilieae	*Trichilia lepidota*	41
Feroniellide C	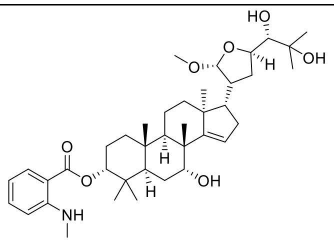	Rutaceae	Aurantioideae	*Feroniella lucida*	42
Chisocheton A	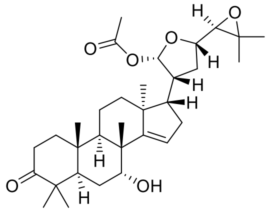	Rutaceae	Zanthoxyloideae	*Vepris uguenensis*	43
Brujavanone L	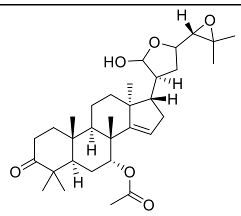	Simaroubaceae		*Brucea javanica*	44
No trivial name	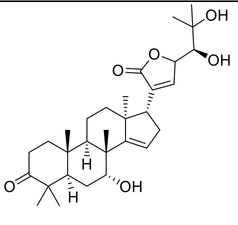	Simaroubaceae		*Picrolemma granatensis*	45

Table S8. Representative examples of glabretanes previously isolated from Meliaceae, Rutaceae, and Simaroubaceae plants out of ca. 110 structurally related natural products listed in Reaxys. Stereochemistry is shown as reported in the literature.

Trivial name	Structure	Family	Subfamily	Species	Reference
7-Deacetylglabretal-3-acetate	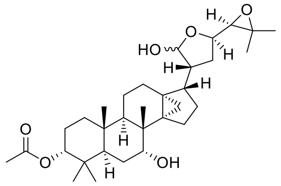	Meliaceae	Aglaieae	*Aglaia ferruginaea*	46
No trivial name	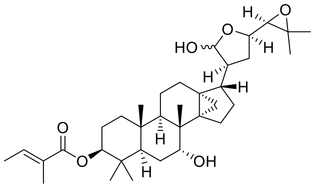	Meliaceae	Aglaieae	*Aglaia crassinervia*	47
No trivial name	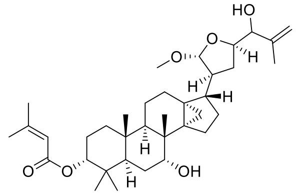	Meliaceae	Cedreleae	*Cedrela sinensis*	48
Glabretal	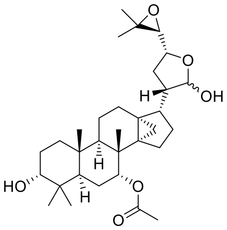	Meliaceae	Melioideae	*Guarea glabra*	49
Cumingianoside F	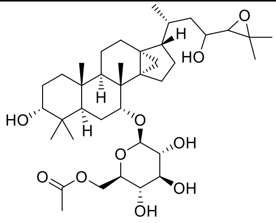	Meliaceae	Melioideae	*Dysoxylum cumingianum*	50
Cumingianoside D	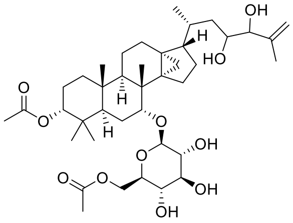	Meliaceae	Melioideae	*Dysoxylum cumingianum*	50

No trivial name	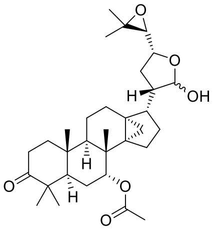	Meliaceae	Melioideae	*Guarea glabra*	49
Dysoxylic acid B	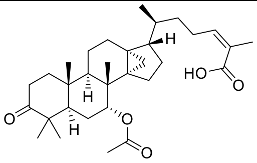	Meliaceae	Melioideae	*Dysoxylum pettigrewianum*	51
Dysoxin 2B	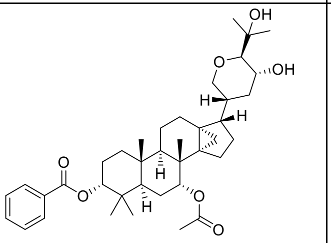	Meliaceae	Melioideae	*Dysoxylum muelleri*	52
3-Episkimmiarepin A	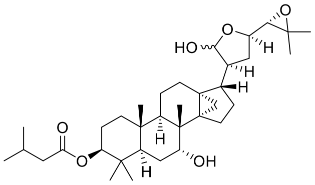	Rutaceae	Aurantioideae	*Luvunga sarmentosa*	53
Skimmiarepin A	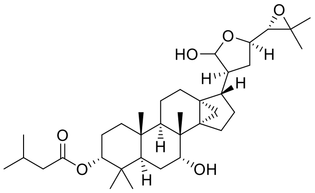	Rutaceae	Aurantioideae	*Aegle marmelos*	54
		Rutaceae	Aurantioideae	*Skimmia japonica*	55
No trivial name	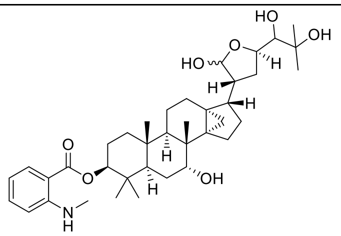	Rutaceae	Zanthoxyloideae	*Raulinoa echinata*	56
3-Oxoskimmiarepin	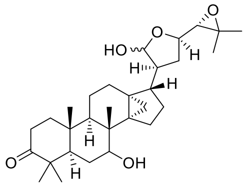	Rutaceae	Zanthoxyloideae	*Zanthoxylum petiolare*	57

Ailanthol	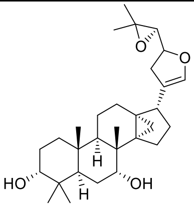	Simaroubaceae		*Ailanthus malabarica*	58,59
Ailanthusin C	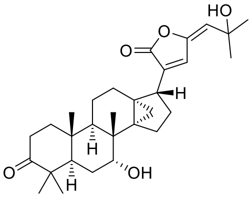	Simaroubaceae		*Ailanthus triphysa*	60

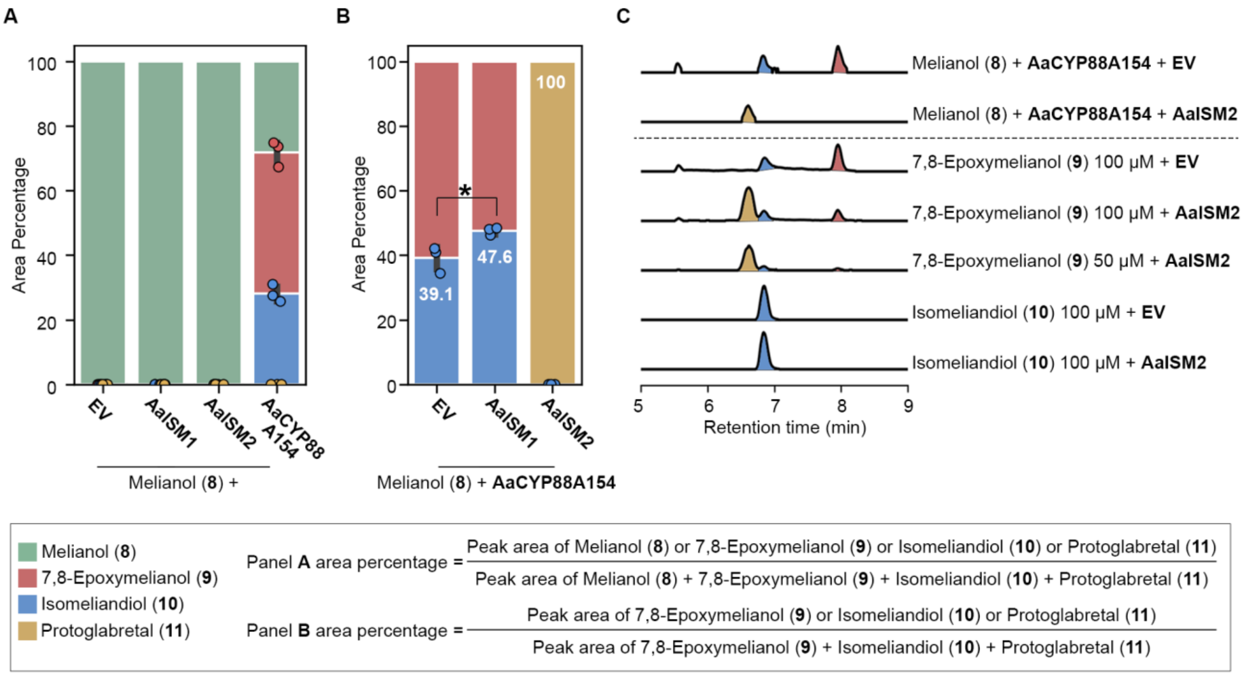

Figure S30. *In vitro* activity of AaCYP88A154, AaISM1 and AaISM2 produced in *Saccharomyces cerevisiae* microsomes is consistent with activity in *Nicotiana benthamiana*. (A) Oxidation of melianol (**8**) by AaCYP88A154 leads to formation of 7,8-epoxymelianol (**9**) and the rearranged product isomeliandiol (**10**). Microsomes additionally contained *Arabidopsis thaliana* cytochrome P450 reductase (CPR).[17] (B) AaISM1 causes a statistically significant increase of isomeliandiol (**10**) peak area compared to 7,8-epoxymelianol (**9**) peak area, whereas AaISM2 completely converts 7,8-epoxymelianol (**9**) to protoglabretal (**11**). The asterisk indicates the significant difference between EV and ISM1 samples using a *t* test (two tailed, equal variance, p = 0.0275, n = 3). Error bars indicate ± standard deviation. 7,8-Epoxymelianol (**9**) was generated *in situ* from microsomes enriched with AaCYP88A154 and *Arabidopsis thaliana* cytochrome P450 reductase (CPR). (C) ISM2 accepts 7,8-epoxymelianol (**9**) generated enzymatically (lanes 1-2) or synthetically (lanes 3-5) as a substrate, but not isomeliandiol (**10**) (lanes 6-7). Comparable results were observed with three replicates from three independent experiments. All chromatograms and area integrations for the stacked bar plots are based on extracted ion chromatograms at mass 511 ($[M+Na]^+$ for **9**, **10** and **11**; $[M+K]^+$ for **8**).

Ergosterol biosynthesis (fungi):

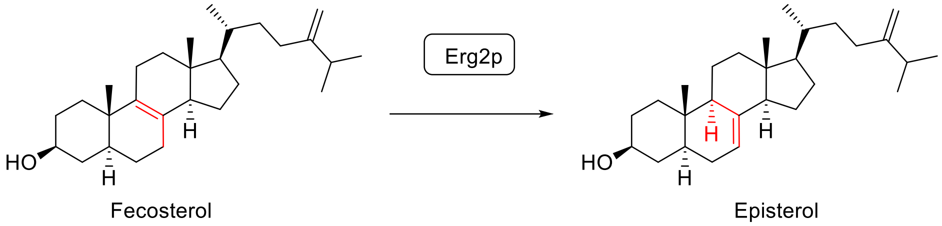

Cholesterol biosynthesis (animals):

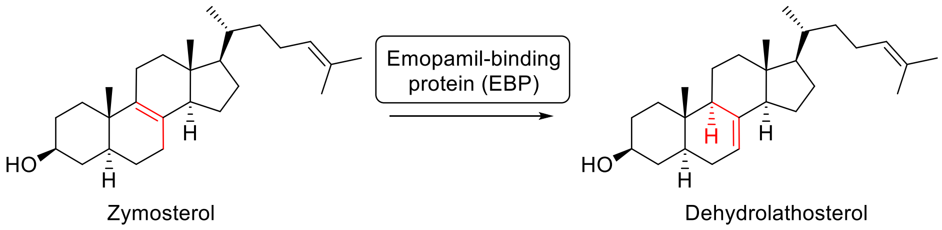

Phytosterol biosynthesis (plants):

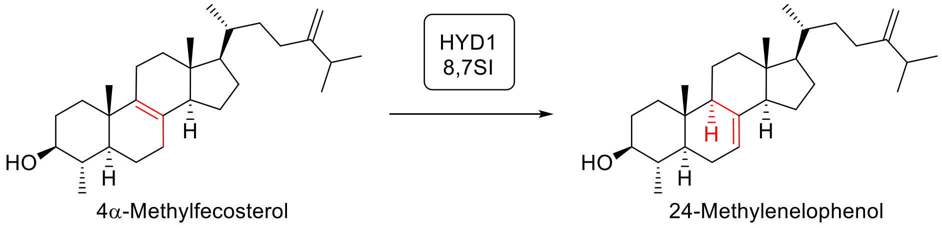

Figure S31. Overview over common C-8,7 sterol isomerase (8,7SI) reactions in primary metabolism.

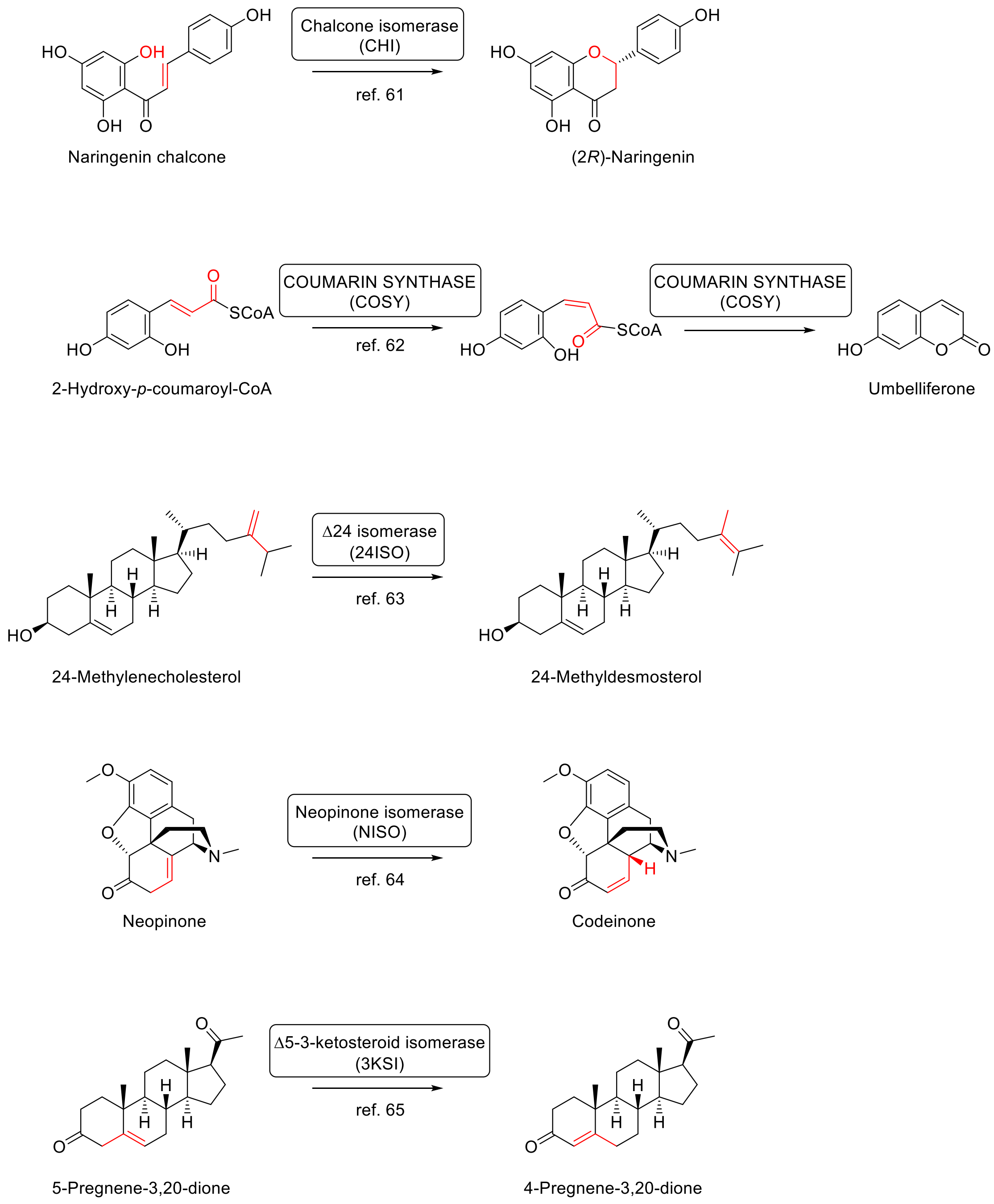

Figure S32. Overview over other isomerase-catalyzed reactions in plant specialized metabolism.[61–65]

References

(1) Chuang, L.; Liu, S.; Biedermann, D.; Franke, J. Identification of Early Quassinoid Biosynthesis in the Invasive Tree of Heaven (*Ailanthus Altissima*) Confirms Evolutionary Origin from Protolimonoids. *Front. Plant Sci.* **2022**, *13*, 958138.

(2) Bally, J.; Jung, H.; Mortimer, C.; Naim, F.; Philips, J. G.; Hellens, R.; Bombarely, A.; Goodin, M. M.; Waterhouse, P. M. The Rise and Rise of *Nicotiana Benthamiana*: A Plant for All Reasons. *Annu. Rev. Phytopathol.* **2018**, *56* (1), 405–426. https://doi.org/10.1146/annurev-phyto-080417-050141.

(3) Chuang, L.; Franke, J. Rapid Combinatorial Coexpression of Biosynthetic Genes by Transient Expression in the Plant Host Nicotiana Benthamiana. In *Engineering Natural Product Biosynthesis: Methods and Protocols*; Skellam, E., Ed.; Methods in Molecular Biology; Springer US: New York, NY, 2022; pp 395–420. https://doi.org/10.1007/978-1-0716-2273-5_20.

(4) Haas, B. J.; Papanicolaou, A.; Yassour, M.; Grabherr, M.; Blood, P. D.; Bowden, J.; Couger, M. B.; Eccles, D.; Li, B.; Lieber, M.; MacManes, M. D.; Ott, M.; Orvis, J.; Pochet, N.; Strozzi, F.; Weeks, N.; Westerman, R.; William, T.; Dewey, C. N.; Henschel, R.; LeDuc, R. D.; Friedman, N.; Regev, A. De Novo Transcript Sequence Reconstruction from RNA-Seq Using the Trinity Platform for Reference Generation and Analysis. *Nat. Protoc.* **2013**, *8* (8), 1494–1512. https://doi.org/10.1038/nprot.2013.084.

(5) Patro, R.; Duggal, G.; Love, M. I.; Irizarry, R. A.; Kingsford, C. Salmon Provides Fast and Bias-Aware Quantification of Transcript Expression. *Nat. Methods* **2017**, *14* (4), 417–419. https://doi.org/10.1038/nmeth.4197.

(6) Robinson, M. D.; Oshlack, A. A Scaling Normalization Method for Differential Expression Analysis of RNA-Seq Data. *Genome Biol.* **2010**, *11* (3), R25. https://doi.org/10.1186/gb-2010-11-3-r25.

(7) Wehrens, R.; Buydens, L. M. C. Self- and Super-Organizing Maps in R: The Kohonen Package. *J. Stat. Softw.* **2007**, *21*, 1–19. https://doi.org/10.18637/jss.v021.i05.

(8) Payne, R. M. E.; Xu, D.; Foureau, E.; Teto Carqueijeiro, M. I. S.; Oudin, A.; de Bernonville, T. D.; Novak, V.; Burow, M.; Olsen, C.-E.; Jones, D. M.; Tatsis, E. C.; Pendle, A.; Halkier, B. A.; Geu-Flores, F.; Courdavault, V.; Nour-Eldin, H. H.; O'Connor, S. E. An NPF Transporter Exports a Central Monoterpene Indole Alkaloid Intermediate from the Vacuole. *Nat. Plants* **2017**, *3*, 16208. https://doi.org/10.1038/nplants.2016.208.

(9) *Home · TransDecoder/TransDecoder Wiki*. GitHub. https://github.com/TransDecoder/TransDecoder (accessed 2021-10-18).

(10) Mistry, J.; Finn, R. D.; Eddy, S. R.; Bateman, A.; Punta, M. Challenges in Homology Search: HMMER3 and Convergent Evolution of Coiled-Coil Regions. *Nucleic Acids Res.* **2013**, *41* (12), e121. https://doi.org/10.1093/nar/gkt263.

(11) Mistry, J.; Chuguransky, S.; Williams, L.; Qureshi, M.; Salazar, G. A.; Sonnhammer, E. L. L.; Tosatto, S. C. E.; Paladin, L.; Raj, S.; Richardson, L. J.; Finn, R. D.; Bateman, A. Pfam: The Protein Families Database in 2021. *Nucleic Acids Res.* **2021**, *49* (D1), D412–D419. https://doi.org/10.1093/nar/gkaa913.

(12) Sainsbury, F.; Thuenemann, E. C.; Lomonossoff, G. P. PEAQ: Versatile Expression Vectors for Easy and Quick Transient Expression of Heterologous Proteins in Plants. *Plant Biotechnol. J.* **2009**, *7* (7), 682–693. https://doi.org/10.1111/j.1467-7652.2009.00434.x.

(13) Peyret, H.; Brown, J. K. M.; Lomonossoff, G. P. Improving Plant Transient Expression through the Rational Design of Synthetic 5′ and 3′ Untranslated Regions. *Plant Methods* **2019**, *15* (1), 108. https://doi.org/10.1186/s13007-019-0494-9.

(14) Reed, J.; Stephenson, M. J.; Miettinen, K.; Brouwer, B.; Leveau, A.; Brett, P.; Goss, R. J. M.; Goossens, A.; O'Connell, M. A.; Osbourn, A. A Translational Synthetic Biology Platform for Rapid Access to Gram-Scale Quantities of Novel Drug-like Molecules. *Metab. Eng.* **2017**, *42*, 185–193. https://doi.org/10.1016/j.ymben.2017.06.012.

(15) Horn, A.; Kazmaier, U. Purified MCPBA, a Useful Reagent for the Oxidation of Aldehydes. *Eur. J. Org. Chem.* **2018**, *2018* (20–21), 2531–2536. https://doi.org/10.1002/ejoc.201701645.

(16) Gietz, R. D.; Schiestl, R. H. High-Efficiency Yeast Transformation Using the LiAc/SS Carrier DNA/PEG Method. *Nat. Protoc.* **2007**, *2* (1), 31–34. https://doi.org/10.1038/nprot.2007.13.

(17) Urban, P.; Mignotte, C.; Kazmaier, M.; Delorme, F.; Pompon, D. Cloning, Yeast Expression, and Characterization of the Coupling of Two Distantly Related *Arabidopsis Thaliana* NADPH-Cytochrome P450 Reductases with P450 CYP73A5. *J. Biol. Chem.* **1997**, *272* (31), 19176–19186. https://doi.org/10.1074/jbc.272.31.19176.

(18) Grebenok, R. J.; Ohnmeiss, T. E.; Yamamoto, A.; Huntley, E. D.; Galbraith, D. W.; Della Penna, D. Isolation and Characterization of an Arabidopsis Thaliana C-8,7 Sterol Isomerase: Functional and Structural Similarities to Mammalian C-8,7 Sterol Isomerase/Emopamil-Binding Protein. *Plant Mol. Biol.* **1998**, *38* (5), 807–815. https://doi.org/10.1023/A:1006028623875.

(19) Xu, Q.; Chen, L.-L.; Ruan, X.; Chen, D.; Zhu, A.; Chen, C.; Bertrand, D.; Jiao, W.-B.; Hao, B.-H.; Lyon, M. P.; Chen, J.; Gao, S.; Xing, F.; Lan, H.; Chang, J.-W.; Ge, X.; Lei, Y.; Hu, Q.; Miao, Y.; Wang, L.; Xiao, S.; Biswas, M. K.; Zeng, W.; Guo, F.; Cao, H.; Yang, X.; Xu, X.-W.; Cheng, Y.-J.; Xu, J.; Liu, J.-H.; Luo, O. J.; Tang, Z.; Guo, W.-W.; Kuang, H.; Zhang, H.-Y.; Roose, M. L.; Nagarajan, N.; Deng, X.-X.; Ruan, Y. The Draft Genome of Sweet Orange (Citrus Sinensis). *Nat. Genet.* **2013**, *45* (1), 59–66. https://doi.org/10.1038/ng.2472.

(20) Wang, X.; Xu, Y.; Zhang, S.; Cao, L.; Huang, Y.; Cheng, J.; Wu, G.; Tian, S.; Chen, C.; Liu, Y.; Yu, H.; Yang, X.; Lan, H.; Wang, N.; Wang, L.; Xu, J.; Jiang, X.; Xie, Z.; Tan, M.; Larkin, R. M.; Chen, L.-L.; Ma, B.-G.; Ruan, Y.; Deng, X.; Xu, Q. Genomic Analyses of Primitive, Wild and Cultivated Citrus Provide Insights into Asexual Reproduction. *Nat. Genet.* **2017**, *49* (5), 765–772. https://doi.org/10.1038/ng.3839.

(21) Ji, Y.-T.; Xiu, Z.; Chen, C.-H.; Wang, Y.; Yang, J.-X.; Sui, J.-J.; Jiang, S.-J.; Wang, P.; Yue, S.-Y.; Zhang, Q.-Q.; Jin, J.; Wang, G.-S.; Wei, Q.-Q.; Wei, B.; Wang, J.; Zhang, H.-L.; Zhang, Q.-Y.; Liu, J.; Liu, C.-J.; Jian, J.-B.; Qu, C.-Q. Long Read Sequencing of Toona Sinensis (A. Juss) Roem: A Chromosome-Level Reference Genome for the Family Meliaceae. *Mol. Ecol. Resour.* **2021**, *21* (4), 1243–1255. https://doi.org/10.1111/1755-0998.13318.

(22) Wang, P.; Luo, Y.; Huang, J.; Gao, S.; Zhu, G.; Dang, Z.; Gai, J.; Yang, M.; Zhu, M.; Zhang, H.; Ye, X.; Gao, A.; Tan, X.; Wang, S.; Wu, S.; Cahoon, E. B.; Bai, B.; Zhao, Z.; Li, Q.; Wei, J.; Chen, H.; Luo, R.; Gong, D.; Tang, K.; Zhang, B.; Ni, Z.; Huang, G.; Hu, S.; Chen, Y. The Genome Evolution and Domestication of Tropical Fruit Mango. *Genome Biol.* **2020**, *21* (1), 60. https://doi.org/10.1186/s13059-020-01959-8.

(23) Yang, J.; Wariss, H. M.; Tao, L.; Zhang, R.; Yun, Q.; Hollingsworth, P.; Dao, Z.; Luo, G.; Guo, H.; Ma, Y.; Sun, W. De Novo Genome Assembly of the Endangered Acer Yangbiense, a Plant Species with Extremely Small Populations Endemic to Yunnan Province, China. *GigaScience* **2019**, *8* (7), giz085. https://doi.org/10.1093/gigascience/giz085.

(24) Rahier, A.; Pierre, S.; Riveill, G.; Karst, F. Identification of Essential Amino Acid Residues in a Sterol 8,7-Isomerase from Zea Mays Reveals Functional Homology and Diversity with the Isomerases of Animal and Fungal Origin. *Biochem. J.* **2008**, *414* (2), 247–259. https://doi.org/10.1042/BJ20080292.

(25) Kikuchi, S.; Satoh, K.; Nagata, T.; Kawagashira, N.; Doi, K.; Kishimoto, N.; Yazaki, J.; Ishikawa, M.; Yamada, H.; Ooka, H.; Hotta, I.; Kojima, K.; Namiki, T.; Ohneda, E.; Yahagi, W.; Suzuki, K.; Li, C. J.; Ohtsuki, K.; Shishiki, T.; Otomo, Y.; Murakami, K.; Iida, Y.; Sugano, S.; Fujimura,

T.; Suzuki, Y.; Tsunoda, Y.; Kurosaki, T.; Kodama, T.; Masuda, H.; Kobayashi, M.; Xie, Q.; Lu, M.; Narikawa, R.; Sugiyama, A.; Mizuno, K.; Yokomizo, S.; Niikura, J.; Ikeda, R.; Ishibiki, J.; Kawamata, M.; Yoshimura, A.; Miura, J.; Kusumegi, T.; Oka, M.; Ryu, R.; Ueda, M.; Matsubara, K.; Kawai, J.; Carninci, P.; Adachi, J.; Aizawa, K.; Arakawa, T.; Fukuda, S.; Hara, A.; Hashidume, W.; Hayatsu, N.; Imotani, K.; Ishii, Y.; Itoh, M.; Kagawa, I.; Kondo, S.; Konno, H.; Miyazaki, A.; Osato, N.; Ota, Y.; Saito, R.; Sasaki, D.; Sato, K.; Shibata, K.; Shinagawa, A.; Shiraki, T.; Yoshino, M.; Hayashizaki, Y. Collection, Mapping, and Annotation of Over 28,000 CDNA Clones from Japonica Rice. *Science* **2003**, *301* (5631), 376–379. https://doi.org/10.1126/science.1081288.

(26) Bae, S.-H.; Lee, J. N.; Fitzky, B. U.; Seong, J.; Paik, Y.-K. Cholesterol Biosynthesis from Lanosterol: Molecular Cloning, Tissue Distribution, Expression, Chromosomal Localization, and Regulation of Rat 7-Dehydrocholesterol Reductase, a Smith-Lemli-Opitz Syndrome-Related Protein. *J. Biol. Chem.* **1999**, *274* (21), 14624–14631. https://doi.org/10.1074/jbc.274.21.14624.

(27) Silve, S.; Dupuy, P. H.; Labit-Lebouteiller, C.; Kaghad, M.; Chalon, P.; Rahier, A.; Taton, M.; Lupker, J.; Shire, D.; Loison, G. Emopamil-Binding Protein, a Mammalian Protein That Binds a Series of Structurally Diverse Neuroprotective Agents, Exhibits Δ8-Δ7 Sterol Isomerase Activity in Yeast *. *J. Biol. Chem.* **1996**, *271* (37), 22434–22440. https://doi.org/10.1074/jbc.271.37.22434.

(28) Hanner, M.; Moebius, F. F.; Weber, F.; Grabner, M.; Striessnig, J.; Glossmann, H. Phenylalkylamine Ca2+ Antagonist Binding Protein: Molecular Cloning, Tissue Distribution, and Heterologous Expression. *J. Biol. Chem.* **1995**, *270* (13), 7551–7557. https://doi.org/10.1074/jbc.270.13.7551.

(29) Long, T.; Hassan, A.; Thompson, B. M.; McDonald, J. G.; Wang, J.; Li, X. Structural Basis for Human Sterol Isomerase in Cholesterol Biosynthesis and Multidrug Recognition. *Nat. Commun.* **2019**, *10* (1), 2452. https://doi.org/10.1038/s41467-019-10279-w.

(30) Yao, H.; Cai, H.; Li, D. Thermostabilization of Membrane Proteins by Consensus Mutation: A Case Study for a Fungal Δ8-7 Sterol Isomerase. *J. Mol. Biol.* **2020**, *432* (18), 5162–5183. https://doi.org/10.1016/j.jmb.2020.02.015.

(31) Edgar, R. C.; Batzoglou, S. Multiple Sequence Alignment. *Curr. Opin. Struct. Biol.* **2006**, *16* (3), 368–373. https://doi.org/10.1016/j.sbi.2006.04.004.

(32) Sievers, F.; Higgins, D. G. Clustal Omega for Making Accurate Alignments of Many Protein Sequences. *Protein Sci.* **2018**, *27* (1), 135–145. https://doi.org/10.1002/pro.3290.

(33) Sievers, F.; Wilm, A.; Dineen, D.; Gibson, T. J.; Karplus, K.; Li, W.; Lopez, R.; McWilliam, H.; Remmert, M.; Söding, J.; Thompson, J. D.; Higgins, D. G. Fast, Scalable Generation of High-Quality Protein Multiple Sequence Alignments Using Clustal Omega. *Mol. Syst. Biol.* **2011**, *7* (1), 539. https://doi.org/10.1038/msb.2011.75.

(34) Guindon, S.; Dufayard, J.-F.; Lefort, V.; Anisimova, M.; Hordijk, W.; Gascuel, O. New Algorithms and Methods to Estimate Maximum-Likelihood Phylogenies: Assessing the Performance of PhyML 3.0. *Syst. Biol.* **2010**, *59* (3), 307–321. https://doi.org/10.1093/sysbio/syq010.

(35) Liu, J.; Yang, S.-P.; Ni, G.; Gu, Y.-C.; Yue, J.-M. Triterpenoids from Aglaia Odorata Var. Microphyllina. *J. Asian Nat. Prod. Res.* **2012**, *14* (10), 929–939. https://doi.org/10.1080/10286020.2012.730698.

(36) Liu, D.; Wang, R.; Xuan, L.; Wang, X.; Li, W. Two New Apotirucallane-Type Triterpenoids from the Pericarp of Toona Sinensis and Their Ability to Reduce Oxidative Stress in Rat Glomerular Mesangial Cells Cultured under High-Glucose Conditions. *Molecules* **2020**, *25* (4), 801. https://doi.org/10.3390/molecules25040801.

(37) Mitsui, K.; Saito, H.; Yamamura, R.; Fukaya, H.; Hitotsuyanagi, Y.; Takeya, K. Apotirucallane and Tirucallane Triterpenoids from *Cedrela Sinensis*. *Chem. Pharm. Bull. (Tokyo)* **2007**, *55* (10), 1442–1447. https://doi.org/10.1248/cpb.55.1442.

(38) Ning, J.; He, H.-P.; Li, S.-F.; Geng, Z.-L.; Fang, X.; Di, Y.-T.; Li, S.-L.; Hao, X.-J. Triterpenoids from the Leaves of Toona Ciliata. *J. Asian Nat. Prod. Res.* **2010**, *12* (6), 448–452. https://doi.org/10.1080/10286020.2010.493329.

(39) Nakanishi, T.; Inada, A.; Nishi, M.; Miki, T.; Hino, R.; Fujiwara, T. The Structure of a New Natural Apotirucallane-Type Triterpene and the Stereochemistry of the Related Terpenes. x-Ray and 13c Nmr Spectral Analyses. *Chem. Lett.* **1986**, *15* (1), 69–72. https://doi.org/10.1246/cl.1986.69.

(40) Kurimoto, S.; Kashiwada, Y.; Lee, K.-H.; Takaishi, Y. Triterpenes and a Triterpene Glucoside from Dysoxylum Cumingianum. *Phytochemistry* **2011**, *72* (17), 2205–2211. https://doi.org/10.1016/j.phytochem.2011.08.002.

(41) Terra, W. D. S.; Vieira, I. J. C.; Braz-Filho, R.; Freitas, W. R. de; Kanashiro, M. M.; Torres, M. C. M. Lepidotrichilins A and B, New Protolimonoids with Cytotoxic Activity from Trichilia Lepidota (Meliaceae). *Molecules* **2013**, *18* (10), 12180–12191. https://doi.org/10.3390/molecules181012180.

(42) Phuwapraisirisan, P.; Sombund, S.; Tip-pyang, S.; Siripong, P. Feroniellides C-E, New Apotirucallane Triterpenoids from the Stem Bark of Feroniella Lucida. *Nat. Prod. Res.* **2013**, *27* (8), 753–760. https://doi.org/10.1080/14786419.2012.698410.

(43) Kiplimo, J. J.; Shahidul Islam, Md.; Koorbanally, N. A. Ring A-Seco Limonoids and Flavonoids from the Kenyan Vepris Uguenensis Engl. and Their Antioxidant Activity. *Phytochemistry* **2012**, *83*, 136–143. https://doi.org/10.1016/j.phytochem.2012.06.025.

(44) Dong, S.-H.; Liu, J.; Ge, Y.-Z.; Dong, L.; Xu, C.-H.; Ding, J.; Yue, J.-M. Chemical Constituents from Brucea Javanica. *Phytochemistry* **2013**, *85*, 175–184. https://doi.org/10.1016/j.phytochem.2012.08.018.

(45) Fo, E. R.; Fernandes, J. B.; Vieira, P. C.; da Silva, M. F. das G. F.; Zukerman-Schpector, J.; Corrêa de Lima, R. M. O.; Nascimento, S. C.; Thomas, W. Protolimonoids and Quassinoids from Picrolemma Granatensis. *Phytochemistry* **1996**, *43* (4), 857–862. https://doi.org/10.1016/0031-9422(96)00348-2.

(46) Mulholland, D. A.; Monkhe, T. V. Two Glabretal-Type Triterpenoids from the Heartwood of Aglaia Ferruginaea. *Phytochemistry* **1993**, *34* (2), 579–580. https://doi.org/10.1016/0031-9422(93)80052-T.

(47) Su, B.-N.; Chai, H.; Mi, Q.; Riswan, S.; Kardono, L. B. S.; Afriastini, J. J.; Santarsiero, B. D.; Mesecar, A. D.; Farnsworth, N. R.; Cordell, G. A.; Swanson, S. M.; Kinghorn, A. D. Activity-Guided Isolation of Cytotoxic Constituents from the Bark of Aglaia Crassinervia Collected in Indonesia. *Bioorg. Med. Chem.* **2006**, *14* (4), 960–972. https://doi.org/10.1016/j.bmc.2005.09.012.

(48) Mitsui, K.; Maejima, M.; Saito, H.; Fukaya, H.; Hitotsuyanagi, Y.; Takeya, K. Triterpenoids from Cedrela Sinensis. *Tetrahedron* **2005**, *61* (44), 10569–10582. https://doi.org/10.1016/j.tet.2005.08.044.

(49) Ferguson, G.; Gunn, P. A.; Marsh, W. C.; McCrindle, R.; Restivo, R.; Connolly, J. D.; Fulke, J. W. B.; Henderson, M. S. Triterpenoids from Guarea Glabra(Meliaceae): A New Skeletal Class Identified by Chemical, Spectroscopic, and X-Ray Evidence. *J. Chem. Soc. Chem. Commun.* **1973**, No. 5, 159–160. https://doi.org/10.1039/C39730000159.

(50) Kashiwada, Y.; Fujioka, T.; Chang, J. J.; Chen, I. S.; Mihashi, K.; Lee, K. H. Anti-Tumor Agents. 136. Cumingianosides A-F, Potent Antileukemic New Triterpene Glucosides, and Cumindysosides A and B, Trisnor- and Tetranortriterpene Glucosides with a 14,18-Cycloapoeuphane-Type Skeleton from Dysoxylum Cumingianum. *J. Org. Chem.* **1992**, *57* (25), 6946–6953. https://doi.org/10.1021/jo00051a050.

(51) Mulholland, D. A.; Nair, J. J. Triterpenoids from Dysoxylum Pettigrewianum. *Phytochemistry* **1994**, *37* (5), 1409–1411. https://doi.org/10.1016/S0031-9422(00)90421-7.

(52) Mulholland, D. A.; Nair, J. J.; Taylor, D. A. H. Glabretal Triterpenoids from Dysoxylum Muelleri. *Phytochemistry* **1996**, *42* (6), 1667–1671. https://doi.org/10.1016/0031-9422(96)00170-7.

(53) Kamperdick, C.; Lien, T. P.; Adam, G.; Sung, T. V. Apotirucallane and Tirucallane Triterpenoids from Luvunga Sarmentosa. *J. Nat. Prod.* **2003**, *66* (5), 675–678. https://doi.org/10.1021/np020458+.

(54) Li, J.; Mahdi, F.; Du, L.; Datta, S.; Nagle, D. G.; Zhou, Y.-D. Mitochondrial Respiration Inhibitors Suppress Protein Translation and Hypoxic Signaling via the Hyperphosphorylation and Inactivation of Translation Initiation Factor EIF2α and Elongation Factor EEF2. *J. Nat. Prod.* **2011**, *74* (9), 1894–1901. https://doi.org/10.1021/np200370z.

(55) Ochi, M.; Tatsukawa, A.; Seki, N.; Kotsuki, H.; Shibata, K. Skimmiarepin A and B, Two New Insect Growth Inhibitory Triterpenoids from Skimmia Japonica Thunb. Var. Intermedia Komatsu f. Repens (Nakai) Hara. *Bull. Chem. Soc. Jpn.* **1988**, *61* (9), 3225–3229. https://doi.org/10.1246/bcsj.61.3225.

(56) Biavatti, M. W.; Vieira, P. C.; da Silva, M. F. G. F.; Fernandes, J. B.; Albuquerque, S. Triterpenoid Constituents of Raulinoa Echinata. *J. Nat. Prod.* **2002**, *65* (4), 562–565. https://doi.org/10.1021/np0103970.

(57) Arruda, M. S. P.; Fernandes, J. B.; Vieira, P. C.; Fatima Das G.F. Da Silva, M.; Pirani, J. R. Protolimonoid and Lignans from Zanthoxylum Petiolare. *Phytochemistry* **1994**, *36* (5), 1303–1306. https://doi.org/10.1016/S0031-9422(00)89656-9.

(58) Joshi, B. S.; Kamat, V. N.; William Pelletier, S.; Go, K.; Bhandary, K. The Structure of Ailanthol, a New Triterpenoid from Ailanthus Malabarica DC. *Tetrahedron Lett.* **1985**, *26* (10), 1273–1276. https://doi.org/10.1016/S0040-4039(00)94868-8.

(59) Hitotsuyanagi, Y.; Ozeki, A.; Choo, C. Y.; Chan, K. L.; Itokawa, H.; Takeya, K. Malabanones A and B, Novel Nortriterpenoids from Ailanthus Malabarica DC. *Tetrahedron* **2001**, *57* (35), 7477–7480. https://doi.org/10.1016/S0040-4020(01)00723-2.

(60) Thongnest, S.; Boonsombat, J.; Prawat, H.; Mahidol, C.; Ruchirawat, S. Ailanthusins A-G and nor-Lupane Triterpenoids from Ailanthus Triphysa. *Phytochemistry* **2017**, *134*, 98–105. https://doi.org/10.1016/j.phytochem.2016.11.007.

(61) Jez, J. M.; Bowman, M. E.; Dixon, R. A.; Noel, J. P. Structure and Mechanism of the Evolutionarily Unique Plant Enzyme Chalcone Isomerase. *Nat. Struct. Biol.* **2000**, *7* (9), 786–791. https://doi.org/10.1038/79025.

(62) Vanholme, R.; Sundin, L.; Seetso, K. C.; Kim, H.; Liu, X.; Li, J.; Meester, B. D.; Hoengenaert, L.; Goeminne, G.; Morreel, K.; Haustraete, J.; Tsai, H.-H.; Schmidt, W.; Vanholme, B.; Ralph, J.; Boerjan, W. COSY Catalyses Trans – Cis Isomerization and Lactonization in the Biosynthesis of Coumarins. *Nat. Plants* **2019**, *5* (10), 1066–1075. https://doi.org/10.1038/s41477-019-0510-0.

(63) Knoch, E.; Sugawara, S.; Mori, T.; Poulsen, C.; Fukushima, A.; Harholt, J.; Fujimoto, Y.; Umemoto, N.; Saito, K. Third DWF1 Paralog in Solanaceae, Sterol Δ24-Isomerase, Branches Withanolide Biosynthesis from the General Phytosterol Pathway. *Proc. Natl. Acad. Sci. U. S. A.* **2018**, *115* (34), E8096–E8103. https://doi.org/10.1073/pnas.1807482115.

(64) Dastmalchi, M.; Chen, X.; Hagel, J. M.; Chang, L.; Chen, R.; Ramasamy, S.; Yeaman, S.; Facchini, P. J. Neopinone Isomerase Is Involved in Codeine and Morphine Biosynthesis in Opium Poppy. *Nat. Chem. Biol.* **2019**, *15* (4), 384–390. https://doi.org/10.1038/s41589-019-0247-0.

(65) Meitinger, N.; Geiger, D.; Augusto, T. W.; Maia de Pádua, R.; Kreis, W. Purification of Δ5-3-Ketosteroid Isomerase from Digitalis Lanata. *Phytochemistry* **2015**, *109*, 6–13. https://doi.org/10.1016/j.phytochem.2014.10.025.
